# TRIM13 reduces cholesterol efflux and increases oxidized LDL uptake leading to foam cell formation and atherosclerosis

**DOI:** 10.1016/j.jbc.2024.107224

**Published:** 2024-03-25

**Authors:** Suresh Govatati, Raj Kumar, Monoranjan Boro, James G. Traylor, A. Wayne Orr, Aldons J. Lusis, Gadiparthi N. Rao

**Affiliations:** 1 Department of Physiology, University of Tennessee Health Science Center, Memphis, Tennessee, USA; 2 Department of Pathology, Louisiana State University Health Science Center, Shreveport, Louisiana, USA; 3 Division of Cardiology, Department of Medicine, University of California, Los Angeles, California, USA

**Keywords:** TRIM13, LXRα/β, ABCA1/G1, cholesterol efflux, foam cell, atherosclerosis

## Abstract

Impaired cholesterol efflux and/or uptake can influence arterial lipid accumulation leading to atherosclerosis. Here, we report that tripartite motif-containing protein 13 (TRIM13), a RING-type E3 ubiquitin ligase, plays a role in arterial lipid accumulation leading to atherosclerosis. Using molecular approaches and KO mouse model, we found that TRIM13 expression was induced both in the aorta and peritoneal macrophages (pMφ) of ApoE^−/−^ mice in response to Western diet (WD) *in vivo*. Furthermore, proatherogenic cytokine interleukin-1β also induced TRIM13 expression both in pMφ and vascular smooth muscle cells. Furthermore, we found that TRIM13 *via* ubiquitination and degradation of liver X receptor (LXR)α/β downregulates the expression of their target genes ABCA1/G1 and thereby inhibits cholesterol efflux. In addition, TRIM13 by ubiquitinating and degrading suppressor of cytokine signaling 1/3 (SOCS1/3) mediates signal transducer and activator of transcription 1 (STAT1) activation, CD36 expression, and foam cell formation. In line with these observations, genetic deletion of TRIM13 by rescuing cholesterol efflux and inhibiting foam cell formation protects against diet-induced atherosclerosis. We also found that while TRIM13 and CD36 levels were increased, LXRα/β, ABCA1/G1, and SOCS3 levels were decreased both in Mφ and smooth muscle cells of stenotic human coronary arteries as compared to nonstenotic arteries. More intriguingly, the expression levels of TRIM13 and its downstream signaling molecules were correlated with the severity of stenotic lesions. Together, these observations reveal for the first time that TRIM13 plays a crucial role in diet-induced atherosclerosis, and that it could be a potential drug target against this vascular lesion.

Atherosclerosis, a multifactorial chronic lipid-driven inflammatory vascular disease, is principally initiated by the accumulation of lipids on the inner walls of arteries, which results in the narrowing and obstruction of blood vessels leading to acute coronary heart disease and death (1, 2). Accumulation of lipids, particularly cholesterol in macrophages and smooth muscle cells of arteries is an essential component in the development of atherosclerotic lesions (3, 4, 5). Various genetic and environmental factors that can inhibit cholesterol efflux or enhance its uptake at cellular level may affect cholesterol homeostasis, which results in the accumulation of cholesterol, particularly low-density lipoprotein (LDL)-cholesterol, in the arteries (1, 6). The trapped LDL in endothelial cells and subendothelial space of arterial intima becomes vulnerable to oxidative modifications by myeloperoxidases, lipoxygenases, and/or reactive oxygen species converting into oxidized LDL (oxLDL), which in turn, triggers endothelial dysfunction and monocyte infiltration (7). The infiltrated monocytes may differentiate into foamy macrophages by uptaking and retaining oxLDL (8). Besides, vascular smooth muscle cells (SMCs) can also uptake oxLDL and form foam cells (9). These foamy cells may secrete proinflammatory cytokines and chemokines that provide a positive feedback loop for continuous generation of macrophage and SMC-derived foam cell formation and lesion progression (8).

In exploring the mechanisms underlying foam cell formation, previous work from our laboratory, as well as others, has shown that proatherogenic cues downregulate ABC transporters such as ATP binding cassette subfamily A member 1 (ABCA1) and subfamily G member 1 (ABCG1) that mediate cholesterol efflux and upregulate scavenger receptors such as cluster of differentiation 36 (CD36) that uptake oxLDL (10, 11, 12). Although several studies have reported the importance of transcriptional regulation of ABC transporters (13) and scavenger receptors (14, 15, 16) by proatherogenic cues, little is known on the role of posttranslational regulation of these molecules in atherogenesis. In this regard, we previously reported that Cullin 3, an E3 ubiquitin ligase, dysregulates cholesterol homeostasis *via* ubiquitinating and degrading ABCA1 and inhibiting cholesterol efflux in response to proatherogenic cues (17). Apart from the posttranslational regulation of ABC transporters, E3 ubiquitin ligases such as membrane-associated RING-CH finger protein 6 (MARCH6) and glycoprotein 78 (GP78) have been reported in ubiquitinating and degrading 3-hydroxy-3-methylglutaryl-coenzyme A reductase levels and decreasing cholesterol biosynthesis (18, 19). Similarly, another E3 ubiquitin ligase, namely inducible degrader of the low-density lipoprotein (IDOL), has been reported to ubiquitinate and degrades LDL receptor in the dysregulation of cholesterol homeostasis (20). Although, various E3 ubiquitin ligases have been shown to be linked directly or indirectly to the regulation of cholesterol homeostasis and/or inflammation, their role in atherosclerosis is unclear (21, 22, 23, 24, 25, 26, 27, 28, 29, 30, 31). To this end, in identifying novel proatherogenic targets, we fed WT and ApoE^−/−^ mice with chow diet (CD) or Western diet (WD) and screened aorta and peritoneal macrophages for expression of several E3 ubiquitin ligases, that were selected based on their involvement in lipoprotein metabolism and/or inflammation (18, 19, 20, 21, 22, 23, 24, 25, 26, 27, 28, 29, 30, 31).

Here, we report for the first time that tripartite motif-containing protein 13 (TRIM13), a RING-type E3 ubiquitin ligase that was highly expressed in WD-fed ApoE^−/−^ mice, acts as a proatherogenic E3 ubiquitin ligase. Specifically, we found that WD as well as proatherogenic cytokine interleukin-1β, (IL-1β) induce TRIM13 expression abundantly and that TRIM13, in turn, mediates ubiquitination and degradation of liver X receptor α/β (LXRα/β) leading to downregulation of their target genes ABCA1/G1 expression and cholesterol efflux. In addition, TRIM13 *via* ubiquitinating and degrading suppressor of cytokine signaling 1/3 (SOCS1/3) mediates signal transducer and activator of transcription 1 (STAT1) activation and CD36 expression leading to oxLDL uptake and foam cell formation. We also found that genetic deletion of TRIM13 ameliorates diet-induced atherosclerosis *via* restoration of cholesterol efflux and inhibition of cholesterol uptake and foam cell formation. Together these results show that TRIM13 plays a crucial role in diet-induced atherosclerosis, and that it could be a potential therapeutic target against this vascular lesion.

## Results

### TRIM13 mediates WD-induced LXRα/β ubiquitination and degradation leading to downregulation of ABCA1/G1 levels in mouse aorta and peritoneal macrophages

**Figure 1:**
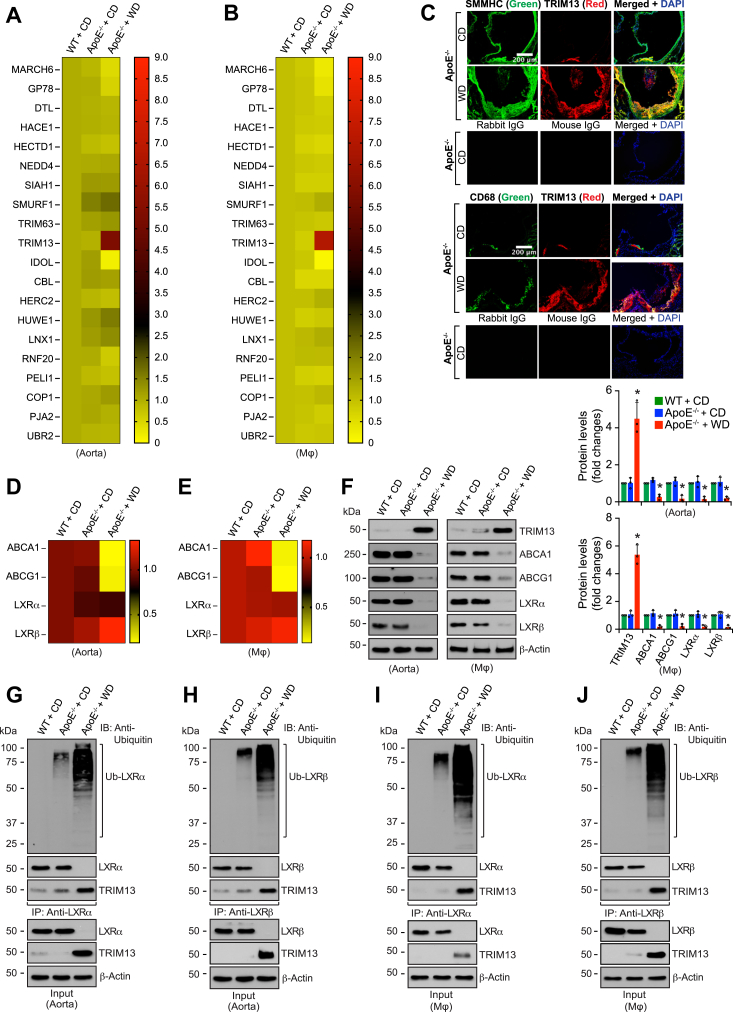
**TRIM13 mediates LXRα/β ubiquitination/degradation leading to downregulation of ABCA1 and ABCG1 levels both in aorta and macrophages.***A* and *B*, heatmaps represent qRT-PCR analysis of RNA from aorta (*A*) and peritoneal macrophages (*B*) of CD-fed WT and CD or 12 weeks of WD-fed ApoE^−/−^ mice for the indicated E3 ubiquitin ligases using their specific primers. The scale represents expression levels of the indicated genes relative to CD-fed WT mice (n = 3). *C*, the aortic root cross sections of CD-fed or 12 weeks of WD-fed ApoE^−/−^ mice were coimmunostained for TRIM13 in combination with SMMHC or CD68, the SMC and macrophage specific markers, respectively (n = 5). *D* and *E*, aorta (*D*) and peritoneal macrophages (*E*) of CD-fed WT and CD or 12 weeks of WD-fed ApoE^−/−^ mice were analyzed by qRT-PCR for the indicated molecules using their specific primers. Heatmaps represent expression levels of the indicated genes relative to CD-fed WT mice (n = 3). *F*, all the conditions were same as in panels (*D* and *E*) expect that aortic or macrophage cell extracts were analyzed by Western blotting for the indicated proteins using their specific antibodies (n = 3). *G*–*J*, equal amounts of protein from aortic (*G* and *H*) and peritoneal macrophage cell (*I* and *J*) extracts from CD-fed WT and CD or 12 weeks of WD-fed ApoE^−/−^ mice were immunoprecipitated with the indicated antibodies and the immunocomplexes were analyzed by Western blotting using anti-ubiquitin antibody. The blots were reprobed for the antigen of the antibody used for immunoprecipitation or TRIM13. The input protein was analyzed by Western blotting for β-actin levels (n = 3). The bar graphs represent mean ± SD values of three independent experiments. The scale bar in panel (*C*) represents 200 μm. ∗*p* < 0.01 *versus* ApoE^−/−^ mice + CD. ABCA1, ATP binding cassette subfamily A member 1; Apo, apolipoprotein; CD, chow diet; LXR, liver X receptor; qRT-PCR, quantitative reverse transcriptase PCR; WD, Western diet; SMC, smooth muscle cell; SMMHC, smooth muscle myosin heavy chain.

To identify novel E3 ubiquitin ligases that are involved in diet-induced atherosclerosis, 8 weeks old WT and ApoE^−/−^ mice were fed with a CD or WD for 12 weeks and the aorta and macrophages of these mice were examined for the expression of several E3 ubiquitin ligases that were linked directly or indirectly to lipoprotein metabolism and/or inflammation (18, 19, 20, 21, 22, 23, 24, 25, 26, 27, 28, 29, 30, 31) by quantitative reverse transcriptase PCR (qRT-PCR). Among the several E3 ubiquitin ligases screened, TRIM13 was found to be induced highly at mRNA level both in aorta and peritoneal macrophages of WD-fed ApoE^−/−^ mice as compared to CD-fed WT or ApoE^−/−^ mice (Fig. 1, *A* and *B*). Besides, we observed that MARCH6, GP78, and IDOL were all decreased both in aorta and peritoneal macrophages of WD-fed ApoE^−/−^ mice as compared to CD-fed WT or ApoE^−/−^ mice (Fig. 1, *A* and *B*). Since the role of MARCH6, GP78, and IDOL in the regulation of cholesterol homeostasis and/or atherosclerosis has been studied previously (18, 19, 20), and very little is known about the role of TRIM13 in vascular diseases, we then focused our attention on this E3 ubiquitin ligase. First, we examined the aortic root cross-sections of CD and 12 weeks of WD-fed ApoE^−/−^ mice for TRIM13 expression in SMCs and macrophages by double immunofluorescence staining. We found that TRIM13 expression increases substantially both in SMCs and macrophages of aortic root of WD-fed ApoE^−/−^ mice as compared to CD-fed ApoE^−/−^ mice (Fig. 1*C*). It was previously reported that TRIM13 ubiquitinates and degrades Nur77, a nuclear receptor, in cancer cells (26). Additionally, Nur77 deficiency has been reported to exacerbate atherosclerosis (32). Based on these clues, we asked whether TRIM13 has any role in the regulation of other nuclear receptors, such as LXRα/β, that play a crucial role in the regulation of cholesterol homeostasis. Therefore, we then studied the effect of WD on the expression levels of LXRα/β and their target genes, ABCA1 and ABCG1. We found that WD decreases LXRα/β only at protein levels but not mRNA levels both in aorta and peritoneal macrophages of ApoE^−/−^ mice as compared to CD-fed WT or ApoE^−/−^ mice (Fig. 1, *D*–*F*). On the other hand, we observed that WD attenuates ABCA1 and ABCG1 both at mRNA and protein levels in aorta as well as peritoneal macrophages of ApoE^−/−^ mice (Fig. 1, *D*–*F*). Since the LXRα/β expression levels were reciprocally correlated with TRIM13 expression at protein but not mRNA levels in WD-fed ApoE^−/−^ mice, we envisioned that these sterol regulatory transcriptional factors might be undergoing ubiquitination and degradation by TRIM13. To test this speculation, the aortic and peritoneal macrophage extracts of CD-fed WT and ApoE^−/−^ mice and 12 weeks WD-fed ApoE^−/−^ mice were immunoprecipitated with LXRα/β antibodies. The immunocomplexes were then analyzed by Western blotting using ubiquitin antibody. The results showed that both LXRα and LXRβ undergo extensive ubiquitination in the aorta and peritoneal macrophages of WD-fed ApoE^−/−^ mice as compared to CD-fed WT or ApoE^−/−^ mice (Fig. 1, *G*–*J*). In addition, reprobing of these blots for TRIM13 showed that TRIM13 interacts with LXRα/β both in aorta and peritoneal macrophages of ApoE^−/−^ mice in response to WD (Fig. 1, *G*–*J*). Moreover, while no differences were observed in LXRα/β levels between WT and ApoE^−/−^ mice on CD, their levels were completely depleted in WD-fed ApoE^−/−^ mice (Fig. 1, *G*–*J*). Together, these findings suggest that TRIM13 may play a role in LXRα/β ubiquitination/degradation, which in turn, may lead to downregulation of their target genes ABCA1 and ABCG1 expression in response to WD.

### TRIM13 mediates IL-1β-induced LXRα/β ubiquitination/degradation leading to downregulation of ABCA1/G1 levels and cholesterol efflux

**Figure 2:**
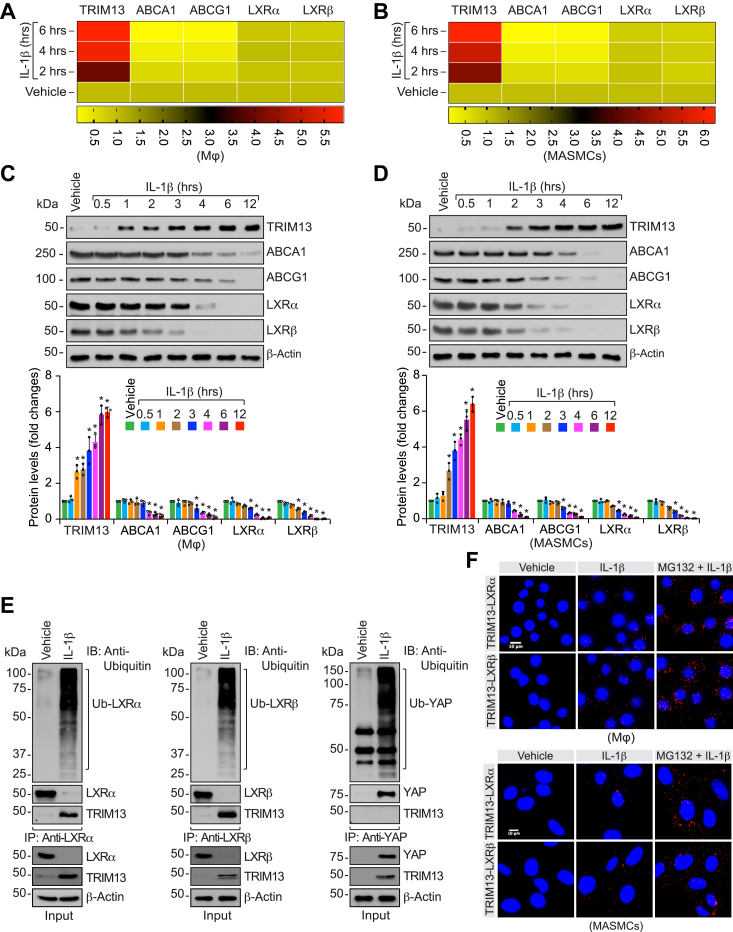
**TRIM13 mediates IL-1β induced LXRα/β ubiquitination/degradation.***A* and *B*, mouse peritoneal macrophages (*A*) or MASMCs (*B*) were treated with and without IL-1β (25 ng/ml) for the indicated time periods; total RNA was isolated and analyzed by qRT-PCR for the indicated genes using their specific primers. Heatmaps represent expression levels of the indicated genes relative to vehicle (n = 3). *C* and *D*, all the conditions were same as in panels (*A* and *B*) expect that cell extracts were analyzed by Western blotting for the indicated proteins using their specific antibodies (n = 3). *E*, mouse peritoneal macrophages were treated with and without IL-1β (25 ng/ml) for 6 h and equal amount of protein from each condition was immunoprecipitated with the indicated antibodies and the immunocomplexes were analyzed by immunoblotting using anti-ubiquitin antibody. The blots were reprobed for the antigen of the antibody used for immunoprecipitation or TRIM13. The input protein was analyzed by Western blotting for β-actin levels (n = 3). *F*, mouse peritoneal macrophages (*upper panel*) and MASMCs (*lower panel*) were treated with and without IL-1β (25 ng/ml) in the presence and absence of MG132 (10 μM) for 6 h and subjected to *in situ* PLA (n = 3). The bar graphs represent mean ± SD values of three independent experiments. The scale bar in panel (*F*) represents 10 μm. ∗*p* < 0.01 *versus* vehicle. IL-1β, interleukin-1β; LXR, liver X receptor; MASMCs, mouse aortic smooth muscle cells; PLA, proximity ligation assay; qRT-PCR, quantitative reverse transcriptase PCR.

To gain further support for the role of TRIM13 in the regulation of LXRα/β levels and their target genes ABCA1 and ABCG1 expression, we next studied the effect of IL-1β, a potent proatherogenic cytokine (33), on mouse peritoneal macrophages and mouse aortic SMCs (MASMCs). IL-1β induced TRIM13 expression with concomitant decrease in ABCA1 and ABCG1 levels in a time-dependent manner both at mRNA and protein levels in peritoneal macrophages and MASMCs (Fig. 2, *A*–*D*). In addition, IL-1β while having no effect on mRNA levels decreased the protein levels of LXRα/β in a time-dependent manner in peritoneal macrophages as well as MASMCs (Fig. 2, *A*–*D*). These results further indicate that TRIM13 might be involved in the ubiquitination and degradation of LXRα/β, thereby affecting their target genes, ABCA1 and ABCG1, expression in response to IL-1β. To test this possibility, we first studied the effect of IL-1β on LXRα/β ubiquitination. Mouse peritoneal macrophages were treated with and without IL-1β for 6 h, cell extracts were prepared, immunoprecipitated with LXRα/β antibodies, and the immunocomplexes were immunoblotted with anti-ubiquitin antibody. As shown in Figure 2*E*, IL-1β induced ubiquitination and degradation of both LXRα/β. In addition, we found that TRIM13 interacts with LXRα/β in response to IL-1β (Fig. 2*E*). Recently, it was reported that IL-1β induces ubiquitination and stabilization of Yes-associated protein (YAP) in promoting atherogenesis (34). Therefore, we also examined TRIM13 role in IL-1β-induced YAP ubiquitination. As expected, IL-1β induced YAP ubiquitination substantially, but no interaction was found between YAP and TRIM13 (Fig. 2*E*). These observations indicate that TRIM13 exhibits substrate specificity. To confirm the interaction between TRIM13 and its substrates, we also performed *in situ* proximity ligation assay (PLA). We found that TRIM13, while having little or no interaction with LXRα/β in control cells, forms complexes with these nuclear receptors in response to IL-1β, and these interactions were further enhanced in the presence of MG132, a potent inhibitor of proteasomal activity (35), in peritoneal macrophages and MASMCs (Fig. 2*F*). These results clearly show that TRIM13 binds with and mediates ubiquitination and degradation of LXRα/β both in macrophages and MASMCs in response to IL-1β. These observations were further confirmed by the findings that downregulation of TRIM13 levels by its siRNA restored LXRα/β and ABCA1/G1 levels from IL-1β-induced downregulation at protein levels both in RAW 264.7 cells and MASMCs (Fig. S1, *A* and *B*). Depletion of TRIM13 levels also restored cholesterol efflux from IL-1β-induced downregulation in both RAW 264.7 cells and MASMCs (Fig. S1, *C* and *D*).

### TRIM13 *via* ubiquitination/degradation of SOCS1/3 levels and activation of STAT1 mediates CD36 expression

**Figure 3:**
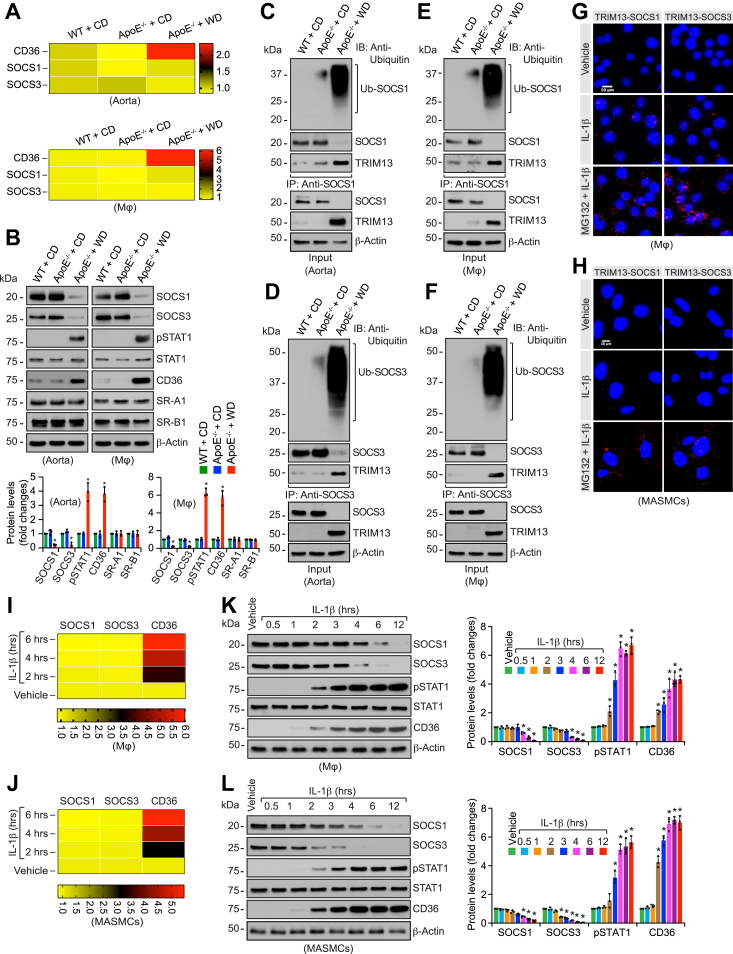
**TRIM13 mediates ubiquitination/degradation of SOCS1/3, activation of STAT1 and induction of CD36 expression in response to WD *in vivo* and IL-1β *in vitro*.***A* and *B*, RNA and protein extracts were prepared from aortas and peritoneal macrophages of CD-fed WT and CD or 12 weeks of WD-fed ApoE^−/−^ mice and analyzed by qRT-PCR (*A*) or Western blotting (*B*), respectively, for the indicated genes and proteins using their specific primers or antibodies, respectively. Heatmaps in panel (*A*) represent expression levels of the indicated genes relative to CD-fed WT mice (n = 3). *C*–*F*, equal amounts of proteins from the extracts of aortas (*C* and *D*) or peritoneal macrophages (*E* and *F*) of CD-fed WT and CD or 12 weeks of WD-fed ApoE^−/−^ mice were immunoprecipitated with the indicated antibodies, and the immunocomplexes were analyzed by Western blotting using anti-ubiquitin antibody. The blots were reprobed for the antigen of the antibody used for immunoprecipitation or TRIM13. The input protein was analyzed by Western blotting for β-actin levels (n = 3). *G* and *H*, mouse peritoneal macrophages (*G*) and MASMCs (*H*) were treated with and without IL-1β (25 ng/ml) in the presence and absence of MG132 (10 μM) for 6 h and subjected to *in situ* PLA (n = 3). *I* and *J*, mouse peritoneal macrophages (*I*) or MASMCs (*J*) were treated with and without IL-1β (25 ng/ml) for the indicated time periods; total RNA was isolated and analyzed by qRT-PCR for the indicated genes using their specific primers. Heatmaps represent expression levels of the indicated genes relative to vehicle (n = 3). *K* and *L*, all the conditions were same as in panels (*I* and *J*) expect that cell extracts were analyzed by Western blotting for the indicated proteins using their specific antibodies (n = 3). The bar graphs represent mean ± SD values of three independent experiments. The scale bars in panel (*G* and *H*) represent 10 μm. ∗*p* < 0.01 *versus* ApoE^−/−^ mice + CD or vehicle. Apo, apolipoprotein; CD, chow diet; CD36, cluster of differentiation 36; IL-1β, interleukin-1β; MASMCs, mouse aortic smooth muscle cells; PLA, proximity ligation assay; SOCS, suppressor of cytokine signaling; STAT1, signal transducer and activator of transcription 1; qRT-PCR, quantitative reverse transcriptase PCR; WD, Western diet.

It was well established that besides the decreased cholesterol efflux, increase in cholesterol uptake may also affect cellular cholesterol levels and influence atherogenesis (36). Therefore, we wanted to test the role of TRIM13 in cholesterol uptake. Previously, we reported that STAT1 *via* CD36 induction, a scavenger receptor for oxLDL uptake, mediates foam cell formation (37). Furthermore, it was demonstrated that SOCS1/3 plays a role in the negative regulation of STAT1 (38). Based on these reports, we wanted to find whether TRIM13 has any impact on the levels of these molecules. In this aspect, WD while downregulating SOCS1/3 only at protein but not at mRNA levels increased STAT1 phosphorylation and CD36 expression both in aorta and peritoneal macrophages of ApoE^−/−^ mice as compared to CD-fed WT or ApoE^−/−^ mice (Fig. 3, *A* and *B*). WD, however, had no major effect on other scavenger receptors such as SR-A1 and SR-B1 in either aorta or peritoneal macrophages. In addition, we found that SOCS1/3 undergoes ubiquitination in response to WD both in aorta and peritoneal macrophages of ApoE^−/−^ mice as compared to CD-fed WT or ApoE^−/−^ mice (Fig. 3, *C*–*F*). Furthermore, TRIM13 was found to be associated with ubiquitinated SOCS1/3 (Fig. 3, *C*–*F*). To confirm the interaction between TRIM13 and SOCS1/3, mouse peritoneal macrophages and MASMCs were treated with and without IL-1β for 6 h and subjected to PLA. TRIM13 while having no association with SOCS1/3 in control cells, showed interaction with these molecules in response to IL-1β, and these interactions were further enhanced in the presence of MG132 both in peritoneal macrophages and MASMCs (Fig. 3, *G* and *H*). These findings reveal that TRIM13 *via* ubiquitination and degradation of SOCS1/3 may lead to STAT1 activation and CD36 expression in response to proatherogenic cues.

### TRIM13 *via* ubiquitination/degradation of SOCS1/3 levels mediates IL-1β-induced STAT1 activation, CD36 expression and foam cell formation

To further validate the above observations, we studied the effect of IL-1β on SOCS1/3, pSTAT1, and CD36 levels both in mouse peritoneal macrophages and MASMCs. We found that IL-1β while having no effect on mRNA levels attenuated SOCS1/3 at protein levels both in peritoneal macrophages and MASMCs (Fig. 3, *I*–*L*). Furthermore, IL-1β stimulated STAT1 phosphorylation and induced CD36 expression at mRNA and protein levels in a time-dependent manner both in peritoneal macrophages and MASMCs (Fig. 3, *I*–*L*). To explore the links between TRIM13, SOCS1/3, pSTAT1, and CD36 levels, we depleted TRIM13 in RAW 264.7 cells and MASMCs by its siRNA and tested its effects on IL-1β-induced SOCS1/3 downregulation, STAT1 phosphorylation, and CD36 expression. Depletion of TRIM13 levels restored SOCS1/3 levels from IL-1β-induced downregulation and suppressed STAT1 phosphorylation and CD36 expression both in RAW 264.7 cells and MASMCs (Fig. S2, *A* and *B*). Depletion of TRIM13 levels also blunted IL-1β-induced foam cell formation both in RAW 264.7 cells and MASMCs (Fig. S2, *C* and *D*). These results indicate that TRIM13 *via* mediating SOCS1/3 ubiquitination and degradation leads to STAT1 activation, CD36 induction and foam cell formation both in macrophages and MASMCs.

### TRIM13 deficiency restores ABCA1/G1 levels and cholesterol efflux from diet-induced downregulation

**Figure 4:**
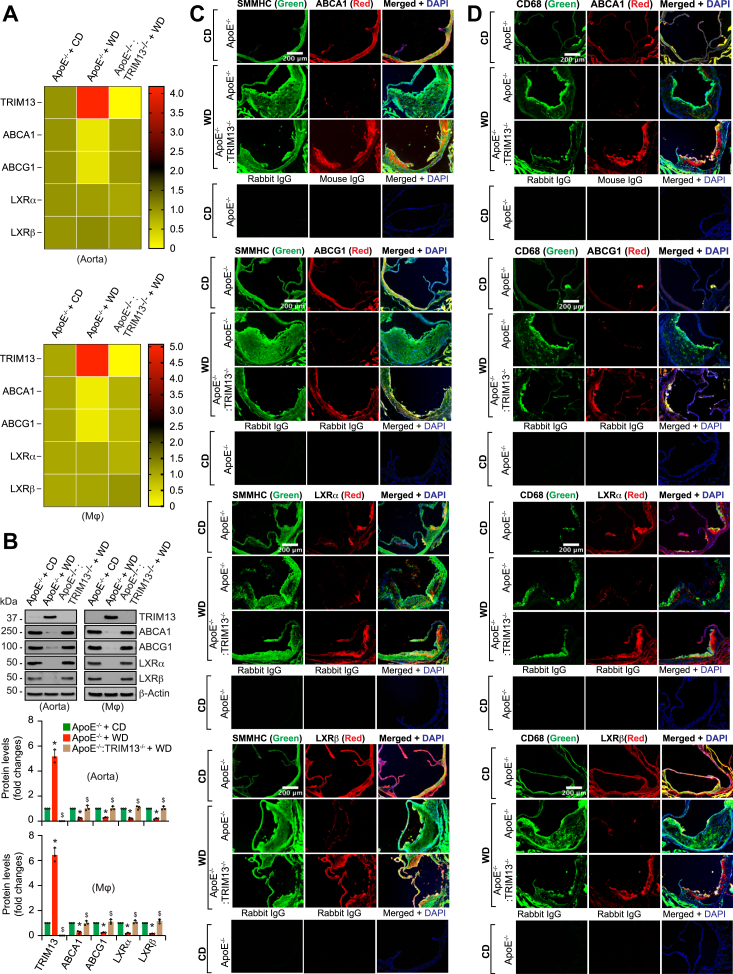

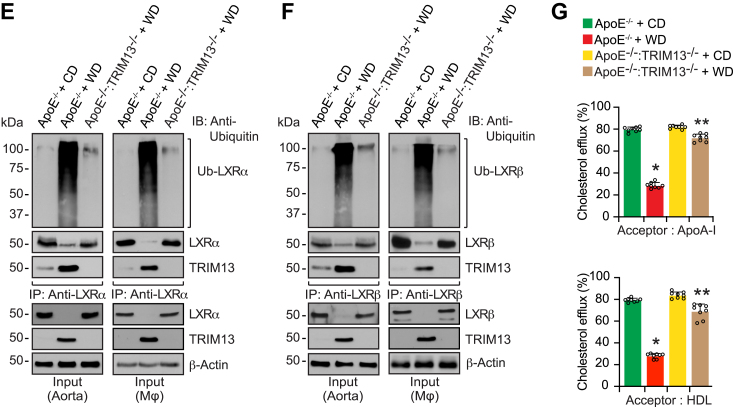
**TRIM13 deficiency restores cholesterol efflux from WD-induced downregulation.***A*, total RNA from aortas and peritoneal macrophages of CD-fed ApoE^−/−^ and 12 weeks of WD-fed ApoE^−/−^ and ApoE^−/−^:TRIM13^−/−^ mice was analyzed by qRT-PCR for the indicated genes using their specific primers. Heatmaps represent expression levels of the indicated genes relative to CD-fed ApoE^−/−^ mice. *B*, all the conditions were same as in panel (*A*) expect that tissue or cell extracts were prepared and analyzed by Western blotting for the indicated proteins using their specific antibodies (n = 3). *C* and *D*, the aortic root cross sections of CD-fed ApoE^−/−^ and 12 weeks of WD-fed ApoE^−/−^ and ApoE^−/−^:TRIM13^−/−^ mice were coimmunostained for ABCA1, ABCG1, LXRα, and LXRβ in combination with SMMHC (*C*) or CD68 (*D*), the SMC and macrophage specific markers, respectively (n = 5). *E* and *F*, equal amounts of proteins from aortic or peritoneal macrophage cell extracts of CD-fed ApoE^−/−^ and 12 weeks of WD-fed ApoE^−/−^ and ApoE^−/−^:TRIM13^−/−^ mice were immunoprecipitated with the indicated antibodies, and the immunocomplexes were analyzed by Western blotting using anti-ubiquitin antibody. The blots were reprobed for the antigen of the antibody used for immunoprecipitation or TRIM13. The input protein was analyzed by Western blotting for β-actin levels (n = 8). *G*, peritoneal macrophages of CD-fed and 12 weeks of WD-fed ApoE^−/−^ and ApoE^−/−^:TRIM13^−/−^ mice were assayed for cholesterol efflux using ApoA-I or HDL (n = 3). The bar graphs represent mean ± SD values of three independent experiments. The scale bar represents 200 μm and 50 μm. ∗*p* < 0.01 *versus* ApoE^−/−^ mice + CD; ^$^*p* < 0.01 *versus* ApoE^−/−^ mice + WD. ABCA1, ATP binding cassette subfamily A member 1; Apo, apolipoprotein; CD, chow diet; HDL, high-density lipoprotein; LXR, liver X receptor; qRT-PCR, quantitative reverse transcriptase PCR; SMMHC, smooth muscle myosin heavy chain; WD, Western diet.

To gain additional support for the role of TRIM13 in the downregulation of cholesterol efflux, we generated ApoE^−/−^:TRIM13^−/−^ mice by cross-breeding ApoE^−/−^ mice with TRIM13^−/−^ mice. The F2 littermates of ApoE^−/−^ and ApoE^−/−^:TRIM13^−/−^ mice were then fed with CD or WD for 12 weeks from the age of 8 weeks and the aortas and peritoneal macrophages were analyzed for cholesterol efflux. Genetic deletion of TRIM13 in ApoE^−/−^ mice restored ABCA1/G1 at both mRNA and protein levels in aorta and peritoneal macrophages from WD-induced downregulation (Fig. 4, *A* and *B*). Regarding LXRα/β levels, TRIM13 deletion while having no effect on mRNA levels rescued LXRα/β protein levels from WD-induced downregulation both in aorta and peritoneal macrophages (Fig. 4, *A* and *B*). No significant differences were observed in LXRα/β and ABCA1/G1 levels between CD-fed ApoE^−/−^ and ApoE^−/−^:TRIM13^−/−^ mice (Fig. S3). We also performed coimmunofluorescence staining of aortic root cross sections of CD-fed ApoE^−/−^ and WD-fed ApoE^−/−^ and ApoE^−/−^:TRIM13^−/−^ mice for ABCA1/G1 and LXRα/β levels in combination with smooth muscle myosin heavy chain (SMMHC) or CD68, specific markers for SMC and macrophages, respectively. WD reduced ABCA1/G1 and LXRα/β levels in SMC and macrophages of only ApoE^−/−^ mice but not ApoE^−/−^:TRIM13^−/−^ mice (Fig. 4, *C* and *D*). Additionally, we found that WD induced ubiquitination and degradation of LXRα/β proteins both in aorta and peritoneal macrophages of ApoE^−/−^ mice as compared to ApoE^−/−^:TRIM13^−/−^ mice (Fig. 4, *E* and *F*). Accordingly, as compared to CD, WD inhibited both ABCA1 and ABCG1-mediated cholesterol efflux in peritoneal macrophages of ApoE^−/−^ mice and TRIM13 deletion restored this effect (Fig. 4*G*).

### TRIM13 deficiency *via* restoring SOCS1/3 levels suppresses diet-induced STAT1 activation, CD36 expression, and foam cell formation

**Figure 5:**
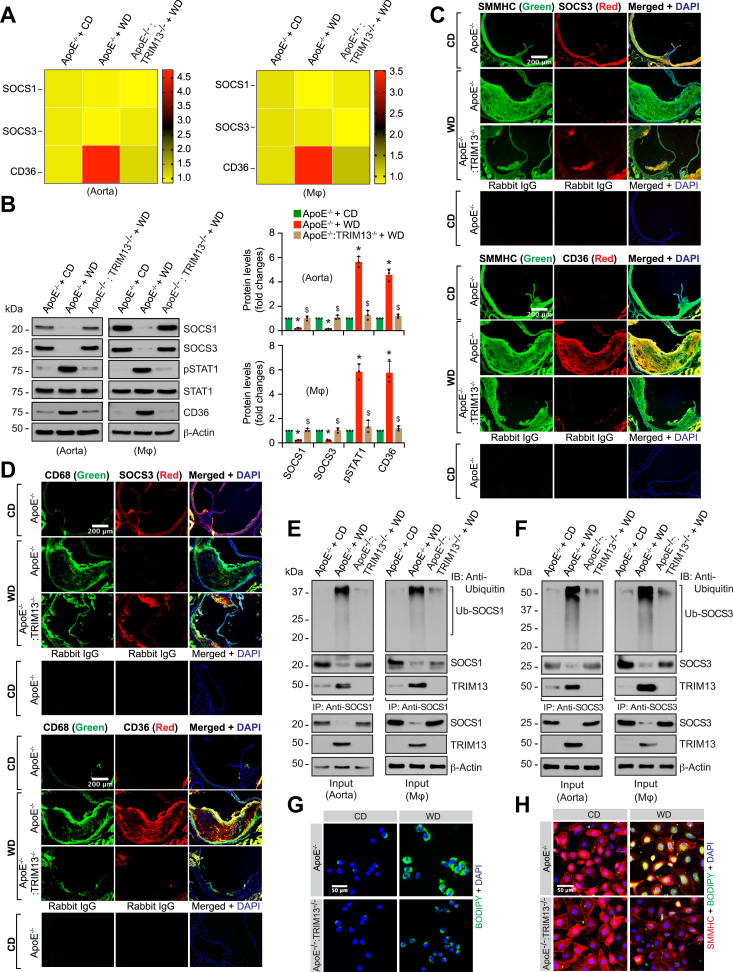
**Genetic deletion of TRIM13 inhibits WD-induced foam cell formation.***A*, total RNA from aortas and peritoneal macrophages of CD-fed ApoE^−/−^ and 12 weeks of WD-fed ApoE^−/−^ and ApoE^−/−^:TRIM13^−/−^ mice was analyzed by qRT-PCR for SOCS1, SOCS3, and CD36 mRNA levels using their specific primers. Heatmaps represent expression levels of the indicated genes relative to CD-fed ApoE^−/−^ mice (n = 3). *B*, all the conditions were same as in panel A expect that tissue or cell extracts were prepared and analyzed by Western blotting for SOCS1, SOCS3, pSTAT1, STAT1, and CD36 levels using their specific antibodies (n = 3). *C* and *D*, the aortic root cross sections of CD-fed ApoE^−/−^ and 12 weeks of WD-fed ApoE^−/−^ and ApoE^−/−^:TRIM13^−/−^ mice were coimmunostained for SOCS3 and CD36 in combination with SMMHC (*C*) or CD68 (*D*), specific markers of SMC and macrophages, respectively (n = 5). *E* and *F*, equal amounts of proteins from aortic and peritoneal macrophage cell extracts of CD-fed ApoE^−/−^ and 12 weeks of WD-fed ApoE^−/−^ and ApoE^−/−^:TRIM13^−/−^ mice were immunoprecipitated with the indicated antibodies, and the immunocomplexes were analyzed by Western blotting using anti-ubiquitin antibody. The blots were reprobed for the antigen of the antibody used for immunoprecipitation or TRIM13. The input protein was analyzed by Western blotting for β-actin levels (n = 3). *G* and *H*, peritoneal macrophages (*G*) or aortic smooth muscle cells (*H*) of CD-fed and 12 weeks of WD-fed ApoE^−/−^ and ApoE^−/−^:TRIM13^−/−^ mice were assayed for foam cell formation using BODIPY staining. Aortic smooth muscle cells were coimmunostained for SMMHC along with BODIPY (n = 3). The bar graphs represent mean ± SD values of three independent experiments. The scale bars in panels (*C* and *D*) represent 200 μm, and in panels (*G* and *H*) are 50 μm. ∗*p* < 0.01 *versus* ApoE^−/−^ mice + CD; ^$^*p* < 0.01 versus ApoE^−/−^ mice + WD. Apo, apolipoprotein; CD, chow diet; CD36, cluster of differentiation 36; qRT-PCR, quantitative reverse transcriptase PCR; SOCS, suppressor of cytokine signaling; SMMHC, smooth muscle myosin heavy chain; STAT1, signal transducer and activator of transcription 1; WD, Western diet.

To understand the role of TRIM13 in cholesterol uptake, we fed ApoE^−/−^ and ApoE^−/−^:TRIM13^−/−^ mice with CD or WD for 12 weeks. We then measured cholesterol uptake. We found that while CD36 levels were increased both at mRNA and protein levels, SOCS1/3 levels were depleted only at protein levels in ApoE^−/−^ mice as compared to ApoE^−/−^:TRIM13^−/−^ mice in response to WD (Fig. 5, *A* and *B*). In regard to STAT1 activation, WD induced STAT1 phosphorylation in ApoE^−/−^ mice as compared to ApoE^−/−^:TRIM13^−/−^ mice both in aorta and peritoneal macrophages (Fig. 5, *A* and *B*). No significant differences were observed in SOCS1/3 and CD36 levels between CD-fed ApoE^−/−^ and ApoE^−/−^:TRIM13^−/−^ mice (Fig. S3) Besides, coimmunofluorescence staining of aortic root cross-sections from CD-fed ApoE^−/−^ and WD-fed ApoE^−/−^ and ApoE^−/−^:TRIM13^−/−^ mice showed downregulation of SOCS3 and upregulation of CD36 both in SMC and macrophages in response to WD in ApoE^−/−^ mice and these effects were diminished in ApoE^−/−^:TRIM13^−/−^ mice (Fig. 5, *C* and *D*). Furthermore, SOCS1/3 were found to be ubiquitinated and degraded in both aorta and peritoneal macrophages of ApoE^−/−^ mice in response to WD and, as expected, these effects were blunted in WD-fed ApoE^−/−^:TRIM13^−/−^ mice (Fig. 5, *E* and *F*). In line with these observations, foam cell formation was increased both in peritoneal macrophages and aortic smooth muscle cells of WD-fed ApoE^−/−^ mice as compared to ApoE^−/−^:TRIM13^−/−^ mice (Fig. 5, *G* and *H*).

### Genetic deletion of TRIM13 gene ameliorates diet-induced atherosclerosis

**Figure 6:**
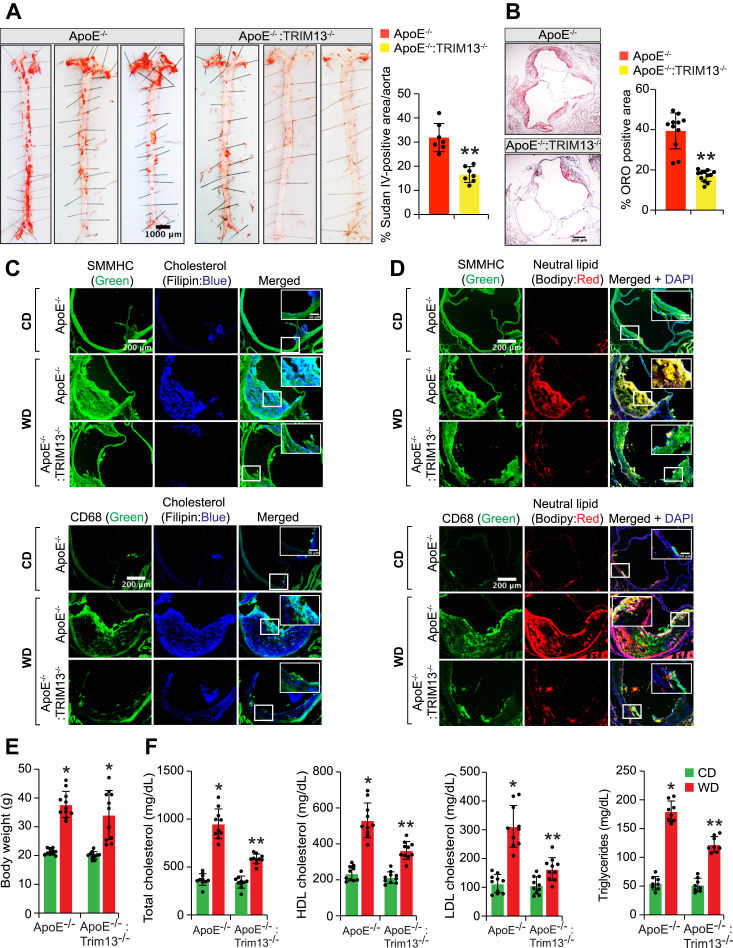
**TRIM13 deletion ameliorates diet-induced atherosclerosis.***A*, representative enface staining pictures of aortas from 12 weeks of WD-fed ApoE^−/−^ and ApoE^−/−^:TRIM13^−/−^ mice. The bar graphs show % of Sudan IV-positive area/aorta (n = 7). *B*–*D*, the aortic root cross sections of 12 weeks of WD-fed ApoE^−/−^ and ApoE^−/−^:TRIM13^−/−^ mice were stained with Oil Red O (*B*) or Filipin (*C*) or BODIPY (*D*) (n = 11 for panel *B* and n = 5 for panels *C* and *D*). *E* and *F*, body weight (*E*) and plasma lipoprotein levels (*F*) of ApoE^−/−^ and ApoE^−/−^:TRIM13^−/−^ mice fed with CD or WD for 12 weeks (n = 10). The bar graphs represent mean ± SD values of three independent experiments. Scale bars in panel B represent 50 μm and panels (*C* and *D*) represent 200 μm. ∗*p* < 0.01 *versus* ApoE^−/−^ + CD; ∗∗*p* < 0.01 *versus* ApoE^−/−^ + WD. Apo, apolipoprotein; CD, chow diet; WD, Western diet.

To support the role of TRIM13 in atherosclerosis, aortas were collected from WD-fed ApoE^−/−^ and ApoE^−/−^:TRIM13^−/−^ mice and analyzed for atherosclerotic lesions by enface staining. Atherosclerotic lesions were substantially decreased in the aortas of ApoE^−/−^:TRIM13^−/−^ mice as compared to ApoE^−/−^ mice on WD (Fig. 6*A*). Besides, the percentage of plaque area in the aortic root cross-sections of ApoE^−/−^:TRIM13^−/−^ mice was also reduced as compared with ApoE^−/−^ mice in response to WD (Fig. 6*B*). These observations were further supported by the staining of the aortic root cross-sections of WD-fed ApoE^−/−^ and ApoE^−/−^:TRIM13^−/−^ mice for filipin and BODIPY (Fig. 6, *C* and *D*). As demonstrated by filipin and BODIPY staining, free cholesterol and neutral lipid levels were substantially reduced in the aortic root cross-sections of ApoE^−/−^ mice as compared to ApoE^−/−^:TRIM13^−/−^ mice in response to WD (Fig. 6, *C* and *D*). To find whether TRIM13 induction affects plasma lipoprotein levels, we collected blood samples from CD and WD-fed ApoE^−/−^ mice and ApoE^−/−^:TRIM13^−/−^ mice and analyzed for plasma lipid levels. No significant differences were noted in the body weight between WD-fed ApoE^−/−^ and ApoE^−/−^:TRIM13^−/−^ mice (Fig. 6*E*). However, total cholesterol, high-density lipoprotein (HDL) cholesterol, LDL cholesterol, and triglyceride levels were all found to be increased in the plasma of WD-fed ApoE^−/−^ mice as compared to CD-fed ApoE^−/−^ mice or WD-fed ApoE^−/−^:TRIM13^−/−^ mice (Fig. 6*F*).

### Severity of human atherosclerosis correlates with heightened expression of TRIM13 levels

**Figure 7:**
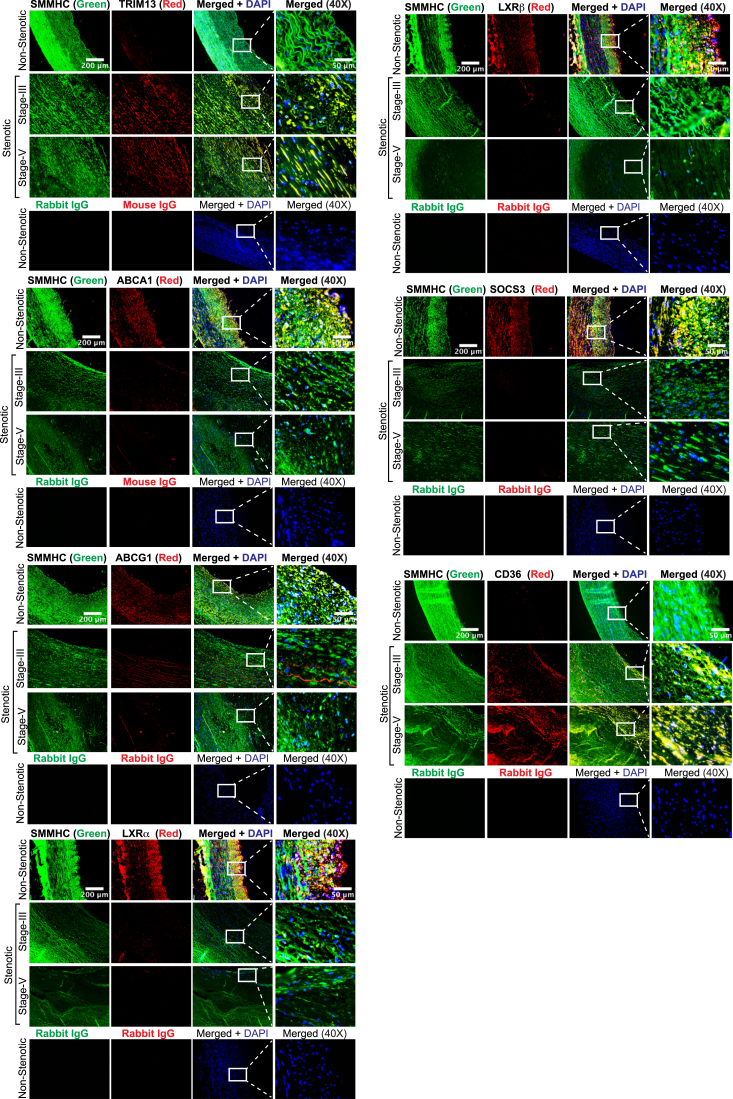

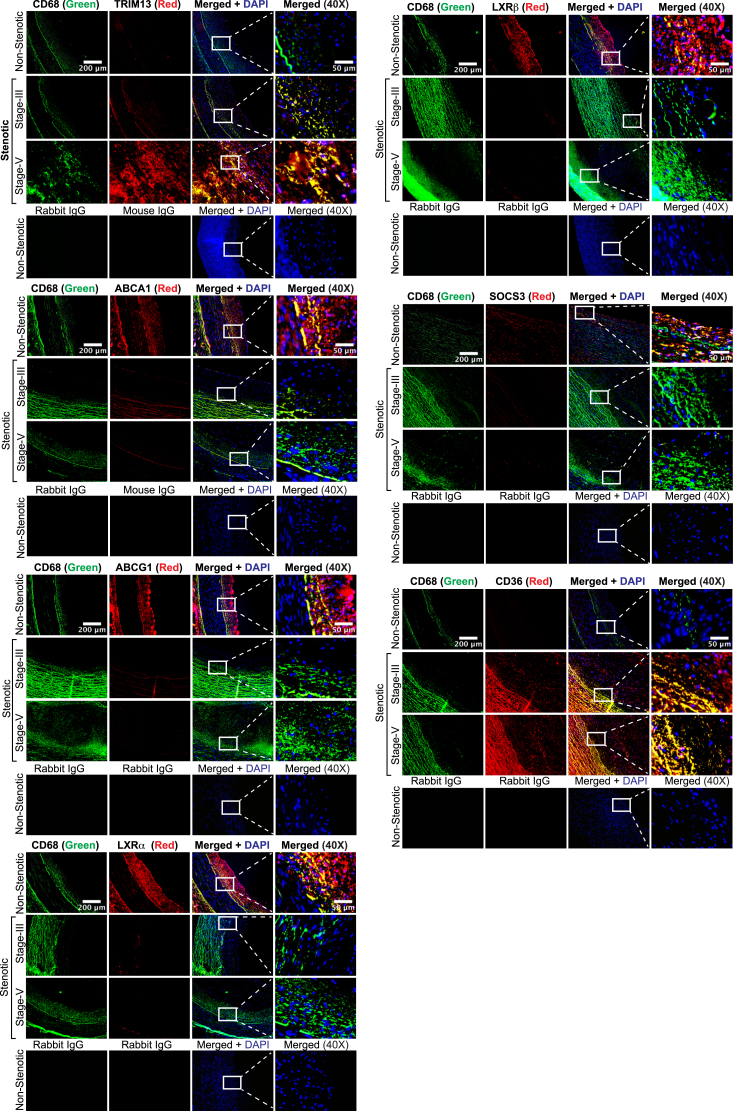
**Upregulation of TRIM13 in human atherosclerosis.** Coimmunofluorescence staining of nonstenotic and stenotic human coronary artery sections for TRIM13, ABCA1, ABCG1, LXRα, LXRβ, SOCS3, and CD36 in combination with SMC- or macrophage-specific markers, SMMHC and CD68, respectively (n = 5). The scale bar represents 200 μm. ABCA1, ATP binding cassette subfamily A member 1; CD36, cluster of differentiation 36; LXR, liver X receptor; SMC, smooth muscle cell; SMMHC, smooth muscle myosin heavy chain; SOCS, suppressor of cytokine signaling.

To validate the translational relevance of the findings obtained in the mouse model of atherosclerosis in humans, we studied the effect of IL-1β on the expression of TRIM13 and its downstream effectors in human cells. As expected, IL-1β induced TRIM13 expression and its induction was correlated with changes in the levels of its downstream effectors both in human aortic smooth muscle cells (HASMCs) and phorbol 12-myristate 13-acetate (PMA)-differentiated human monocytic (THP-1) cells (Fig. S4, *A* and *B*). In addition, siRNA-mediated depletion of TRIM13 levels rescued its downstream effectors from IL-1β-induced changes both in HASMCs and PMA-differentiated THP1 cells (Fig. S4, *A* and *B*). In line with these observations, TRIM13 depletion attenuated IL-1β-induced foam cell formation in HASMCs as well as PMA-differentiated THP1 cells (Fig. S4, *C* and *D*). To further confirm these observations, we coimmunostained nonstenotic and stenotic (stages III and V) human coronary artery cross-sections for TRIM13, ABCA1/G1, LXRα/β, SOCS3, and CD36 in combination with SMMHC or CD68, the SMC and macrophage markers, respectively. We found that while TRIM13 and CD36 levels were increased substantially, ABCA1/G1, LXRα/β, and SOCS3 levels were decreased both in SMC and macrophages of stenotic human coronary artery sections as compared to nonstenotic human coronary artery sections (Fig. 7). Furthermore, the changes in TRIM13, ABCA1/G1, LXRα/β, SOCS1/3, and CD36 levels were correlated with the severity of lesions (Fig. 7).

### Potential role of Sp1 in WD/IL-1β-induced TRIM13 expression

**Figure 8:**
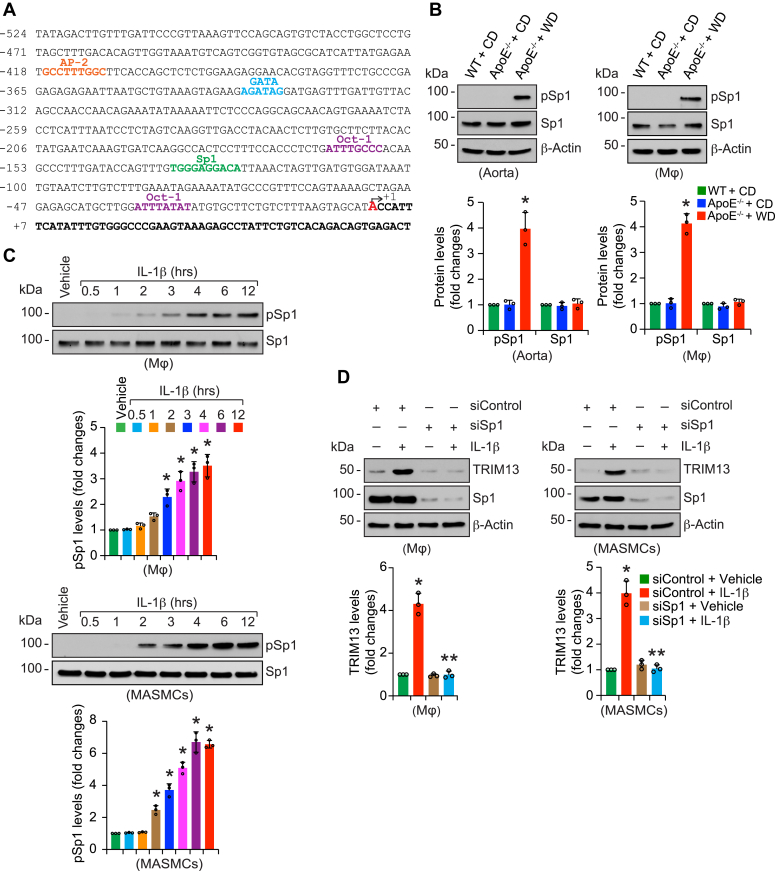
**Sp1 mediates WD/IL-1β-induced TRIM13 expression.***A*, TRANSFAC analysis of mouse TRIM13 promoter sequence for the identification of potential transcription factor binding sites. *B*, protein extracts were prepared from aortas and peritoneal macrophages of CD-fed WT and CD or 12 weeks of WD-fed ApoE^−/−^ mice and analyzed by Western blotting for phospho and total Sp1 levels using their specific antibodies (n = 3). *C*, peritoneal macrophages or MASMCs were treated with and without IL-1β (25 ng/ml) for the indicated time periods, and cell extracts were prepared and analyzed by Western blotting for phospho and total Sp1 levels using their specific antibodies (n = 3). *D*, peritoneal macrophages or MASMCs were transfected with siControl or siSp1 (100 nmoles), and 36 h later, cells were quiesced, treated with and without IL-1β (25 ng/ml) for 6 h, and cell extracts were prepared and analyzed by Western blotting for TRIM13 levels. Cell extracts were also analyzed by Western blotting for Sp1 and β-actin levels to show the efficacy of siRNA on its on-target and off-target molecules (n = 3). The bar graphs represent mean ± SD values of three independent experiments. ∗*p* < 0.01 *versus* CD or Vehicle or siControl + Vehicle; ∗∗*p* < 0.01 *versus* siControl + IL-1β. Apo, apolipoprotein; IL-1β, interleukin-1β; MASMCs, mouse aortic smooth muscle cells; WD, Western diet.

To investigate for possible mechanism by which WD induces TRIM13 expression, we performed TRANSFAC analysis of TRIM13 promoter. TRANSFAC analysis revealed the presence of several Sp1 sites in the TRIM13 promoter (Fig. 8*A*). In addition, several reports showed a role for Sp1 in atherosclerosis (39, 40, 41). Based on these clues, we studied the effect of WD on Sp1 activation. WD induced Sp1 phosphorylation in aorta and peritoneal macrophages of ApoE^−/−^ mice as compared to CD-fed ApoE^−/−^ or WT mice (Fig. 8*B*). Since IL-1β induced TRIM13 expression both in peritoneal macrophages and MASMCs, we next examined for the role of Sp1 in TRIM13 induction by IL-1β. First, IL-1β induced Sp1 phosphorylation both in peritoneal macrophages and MASMCs in a time-dependent manner (Fig. 8*C*). Second, siRNA-mediated depletion of Sp1 levels blocked IL-1β-induced TRIM13 expression both in peritoneal macrophages and MASMCs (Fig. 8*D*).

## Discussion

Progressive accumulation of cholesterol within the inner arterial wall leads to the development and progression of atherosclerotic plaques (1, 2). Decreased cholesterol efflux and increased cholesterol uptake can lead to cholesterol accumulation in macrophages and SMCs of arterial origin and form foam cells (9, 42, 43). Specialized membrane transporters such as ABCA1/G1 play an important role in cholesterol efflux by transferring cellular free cholesterol to plasma apolipoprotein A-I (ApoA-I) and HDL, respectively, from peripheral tissues, which in turn, are transported to the liver and metabolized to bile acids (44, 45, 46, 47, 48). Therefore, dysregulation of these transporters can lead to the accumulation of cholesterol, particularly LDL-cholesterol in the cellular components of the arteries leading to the development of atherosclerosis (49, 50, 51). Previous work from our laboratory as well as others has shown that these ABC transporters undergo proteasomal-mediated degradation affecting cholesterol efflux in response to proatherogenic cues (11, 17, 21). Specifically, we reported that Cullin 3, an E3 ubiquitin ligase, ubiquitinates and degrades ABCA1 in promoting diet-induced atherosclerosis (11, 17). Additionally, the role of other E3 ubiquitin ligases such as HUWE1 and NEDD4-1 in proteasomal degradation of ABCG1/G4 has been reported in other cell types (21). Besides posttranslational regulation, ABC transporters can also be regulated at transcriptional level by proatherogenic cues (13).

In the present study, we demonstrate that TRIM13 regulates ABCA1/G1 at transcriptional level by ubiquitinating and degrading their transcriptional factors, namely, LXRα/β. Our results demonstrate that WD induces TRIM13 expression with concomitant downregulation of LXRα/β at protein level and their target genes, ABCA1/G1, both at mRNA and protein levels in the aortas as well as peritoneal macrophages of ApoE^−/−^ mice. Our findings also show that TRIM13 ubiquitinates and degrades LXRα/β in response to WD in ApoE^−/−^ mice. Like WD, IL-1β, a proinflammatory cytokine (33), also induced the expression of TRIM13 along with the downregulation of LXRα/β at protein level and their target genes, ABCA1/G1, both at mRNA and protein levels in peritoneal macrophages and MASMCs. In addition, TRIM13 interacts with LXRα/β and mediates their ubiquitination and degradation in these cells in response to IL-1β. Furthermore, siRNA-mediated depletion of TRIM13 levels restored LXRα/β and ABCA1/G1 levels and cholesterol efflux from IL-1β-induced downregulation in macrophages and MASMCs. Based on these findings, it appears that TRIM13 *via* ubiquitinating and degrading LXRα/β levels and downregulating the expression of their target genes, ABCA1/G1, suppresses cholesterol efflux leading to foam cell formation.

A large body of evidence shows that accumulation of lipid-laden macrophages and SMCs in the arterial wall triggers plaque formation and atherosclerotic lesion progression (1, 3). In this aspect, besides ABC transporters, scavenger receptors such as CD36, SR-A1, and SR-B1 can also contribute to foam cell formation *via* their ability to bind and internalize oxLDL (9, 52, 53). Previously, others and we have shown a role for CD36 in oxLDL uptake by macrophages and SMCs leading to foam cell formation and atherosclerosis (10, 14, 15, 37). In addition, interaction of CD36 with oxLDL may trigger the production and secretion of many cytokines that could enhance monocyte recruitment leading to accelerated foam cell formation and plaque progression (52). Although several studies have shown a role for transcriptional mechanisms in the regulation of CD36 by various proatherogenic cues (14, 15, 16, 37), the role of E3 ubiquitin ligases in transcriptional induction of CD36 is unknown. To this end, we found an involvement of TRIM13 in transcriptional regulation of CD36. Basically, our observations show that in response to WD, TRIM13 ubiquitinates and degrades SOCS1/3 levels, and this response correlates with STAT1 activation and CD36 induction both in aortas and peritoneal macrophages of ApoE^−/−^ mice. The role of TRIM13 in transcriptional regulation of CD36 can be supported by our findings that IL-1β-induced SOCS1/3 ubiquitination/degradation, STAT1 activation and CD36 expression were dependent on TRIM13. Together, these observations reveal that besides its role in cholesterol efflux, TRIM13 also modulates cholesterol uptake in enhancing foam cell formation.

The genetic deletion of TRIM13 restored WD-induced downregulation of ABCA1/G1 levels and cholesterol efflux *via* suppressing LXRα/β ubiquitination and degradation in ApoE^−/−^ mice. In addition, TRIM13 deficiency blunted WD-induced SOCS1/3 ubiquitination and degradation, thereby attenuating STAT1 activation, CD36 expression and foam cell formation in ApoE^−/−^ mice. Besides these observations, genetic deletion of TRIM13 substantially lowered WD-induced atherosclerotic lesions in the aortic root cross-sections of ApoE^−/−^ mice. Furthermore, we found that the levels of neutral lipids were significantly lower in WD-fed ApoE^−/−^:TRIM13^−/−^ mice as compared to WD-fed ApoE^−/−^ mice; therefore it is likely that, besides its role in cholesterol efflux and uptake, TRIM13 may also be involved in the modulation of cholesterol esterification in response to WD. Since TRIM13 deletion also attenuated plasma lipoprotein levels in WD-fed ApoE^−/−^ mice, it is possible that TRIM13, besides its involvement in curtailing cholesterol efflux and enhancing oxLDL uptake, might be involved in the development of hypercholesterolemia, which, however, needs further investigation. It should be also pointed out that as TRIM13 was induced by both WD and IL-1β in peritoneal macrophages and MASMCs affecting cholesterol efflux and oxLDL uptake, it is likely that both these cell types are contributing to TRIM13-mediated foam cell formation and atherosclerosis. Thus, all these findings clearly demonstrate a role for TRIM13 in diet-induced atherosclerosis.

To extrapolate the translational relevance of our novel findings observed in mouse model system to human atherosclerosis, first, we found induction of TRIM13 and its downstream effectors in HASMCs and PMA-differentiated THP1 cells in response to proatherogenic cue, IL-1β. In addition, we also found that the levels of TRIM13 and CD36 were increased, whereas the levels of ABCA1/G1, LXRα/β, and SOCS3 were decreased both in SMCs and macrophages of stenotic human coronary arteries as compared to nonstenotic coronary arteries. These observations provide an additional support for the role of TRIM13 in the arterial cholesterol accumulation leading to atherosclerosis in humans. Thus, the present study demonstrates that TRIM13 plays an important role in the pathophysiology of atherosclerosis by disrupting cholesterol homeostasis *via* ubiquitinating and degrading LXRα/β, and SOCS1/3 leading to decreased cholesterol efflux and enhanced cholesterol uptake by macrophages and SMCs forming foam cells. Previous studies have shown a role for endothelial cell dysfunction in the initiation of atherosclerosis (54). In this context, it remains to be investigated whether TRIM13 was also involved in endothelial cell dysfunction.

E3 ubiquitin ligases, a crucial component of proteasomes, mediate the transfer of ubiquitin from E2 ubiquitin-conjugating enzymes to substrate proteins leading to their ubiquitination and degradation (55). Thus, recognition of substrates by E3 ubiquitin ligases is a key step in the process of proteasomal-mediated protein degradation (55). Eukaryotic cells express many E3 ubiquitin ligases that facilitate the ubiquitination of a variety of protein substrates in response to various cues (56). A specific E3 ubiquitin ligase can ubiquitinate several proteins (56). Similarly, a specific protein can be ubiquitinated by different E3 ubiquitin ligases (56). Based on these clues, we asked whether TRIM13 has any substrate specificity. To this end, we found that TRIM13 mediates IL-1β-induced ubiquitination of LXRα/β and SOCS1/3 but not YAP in peritoneal macrophages, indicating its substrate specificity. To date, caspase 8, Akt, L-type channels and Nur77 have been reported as substrates of TRIM13 (26, 57, 58, 59). Adding to the list, our present findings show that TRIM13 can also ubiquitinate LXRα/β and SOCS1/3 leading to their degradation, particularly in response to proatherogenic cues such as IL-1β and WD. Based on these observations, it appears that TRIM13 is a hub in affecting cholesterol efflux and oxLDL uptake, particularly under high fat diet-fed conditions, in contributing to atherogenesis.

In regard to the possible mechanism by which WD/IL-1β induces TRIM13 expression, our observations revealed that WD and IL-1β triggers activation of Sp1 *in vivo* in ApoE^−/−^ mice and *in vitro* in peritoneal macrophages and MASMCs, respectively, and depletion of Sp1 levels attenuate IL-1β-induced TRIM13 expression, it is likely that Sp1 plays a role in WD/IL-1β-induced TRIM13 expression. In fact, previous studies have reported a role for Sp1 in experimental atherosclerosis (39, 40, 41). However, as Sp1 binds to GC box motif (60), further studies are required to identify the putative Sp1 binding motif in the TRIM13 promoter mediating TRIM13 induction in response to WD or IL-1β. All these observations reveal for the first time that TRIM13 by affecting cholesterol efflux and oxLDL uptake plays a key role in diet-induced atherosclerosis.

## Experimental procedures

### Materials

Anti-ABCG1 (SC-20795), anti-CD36 (SC-9154 & SC-7309), anti-STAT1 (SC-464), anti-TRIM13 (SC-398129), anti-α-Tubulin (SC-23948), and anti-β-Actin (SC-47778) antibodies were bought from Santa Cruz Biotechnology. Anti-ABCA1 (ab18180), anti-ABCG1 (ab52617), anti-LXRα (ab176323), anti-LXRβ (ab28479), anti-SOCS1 (ab62584), anti-pSp1 (ab59257), Sp1 (ab227383), and anti-SOCS3 (ab280884) antibodies and LDL/VLDL cholesterol assay kit (ab65390) and triglyceride quantification assay kit (ab65336) were obtained from Abcam. Anti-pSTAT1 (7167S) antibody and horseradish peroxidase (HRP)-conjugated light chain specific anti-mouse (58802S) and anti-rabbit immunoglobulin G (IgGs) (93702S) were procured from Cell Signaling Technology. Anti-ubiquitin (P4D1) antibody was purchased from Enzo Life Sciences. Duolink *in situ* PLA kit (DUO92101), IL-1β (I5271), Oil Red O (O0625), and Sudan IV (198102) were obtained from Sigma-Aldrich. PCR Master Mix (M750B) was purchased from Promega. HRP-conjugated goat anti-rabbit IgG (31460) and goat anti-mouse IgG (31437), Lipofectamine 3000 transfection reagent (L3000-015), PowerUp SYBR Green Master Mix (A25780), mouse Sp1 Silencer Select siRNA (s74195), mouse TRIM13 Silencer Select siRNA (s83532), and Silencer Predesigned human TRIM13 siRNA (16838) were obtained from Thermo Fisher Scientific. Control nontargeting siRNA (D-001810-10) was bought from Dharmacon RNAi Technologies. Thioglycolate medium brewer-modified (21176) was obtained from BD Biosciences. Enhanced chemiluminescence Western blotting detection reagents (RPN2106) were purchased from GE HealthCare. All the primers used for qRT-PCR were synthesized by Integrated DNA Technologies and listed in Table S1.

### Animal models

C57BL/6J mice (stock number 000664) and ApoE^−/−^ mice (stock number 002052) were purchased from The Jackson Laboratory. TRIM13^−/−^ mice were obtained from Canadian Mouse Mutant Repository (The Centre for Phenogenomics) at the Hospital of Sick Children. All the mice were maintained at the University of Tennessee Health Science Center’s (UTHSC) laboratory animal care unit. The UTHSC laboratory animal care unit facility maintains a 12/12 h light/dark cycle and the animals had ad libitum access to food and water. ApoE^−/−^:TRIM13^−/−^ mice were generated by crossbreeding ApoE^−/−^ mice with TRIM13^−/−^ mice. ApoE^−/−^:TRIM13^−/−^ mice showed no differences in morphological and behavioral characteristics and reproductive ability as compared to ApoE^−/−^ mice. Mice were bred and maintained according to the institutional animal care and use committee guidelines. To study the role of TRIM13 in diet-induced atherosclerosis, 8-week-old mice (male and female) were kept on either CD (Teklad Irradiated LM-485 mouse/rat diet, Envigo, Catalog number 7912) or WD containing 21% fat and 0.2% cholesterol (Cat # TD.88137, Harlan Teklad, Harlan Laboratories) for 12 weeks. At the end of the experimental period, mice were euthanized by ketamine/xylazine overdose and aorta, peritoneal macrophages and blood were collected for further analysis as required. All the mice used in the study were confirmed by genotyping.

### Ethics statement

All the experiments involving animals were approved by the Institutional Animal Care and Use Committee of the UTHSC. The use of human tissue sections in this study was approved by UTHSC Institutional Review Board.

### Human normal and atherosclerotic artery sections

Collection of human nonstenotic and stenotic coronary arteries, preparation of tissue sections and classification of the lesion grades from I to V were described previously (61). Briefly, the postmortem samples were obtained from both the genders with age group ranging from 21 to 71 years. The plaque severity was determined by H&E staining and scoring by two independent pathologists using a modified Stary classification system (61). These samples were considered to be nonhuman subjects, as they were postmortem samples.

### Isolation of mouse aortic SMCs

Aortic SMCs from CD or WD-fed ApoE^−/−^ and ApoE^−/−^:TRIM13^−/−^ mice were isolated by collagenase II/elastase digestion as per the methods described (62). Isolated SMCs were grown in Dulbecco's modified Eagle's medium (DMEM) medium containing fetal bovine serum (FBS) (10%), penicillin (100 units/ml) and streptomycin (100 μg/ml) at 37 °C in a humidified 95% air and 5% CO_2_ atmosphere. The purity of SMCs was confirmed by staining with anti-SMMHC.

### Isolation of peritoneal macrophages

Mice were injected intraperitoneally with 1 ml of autoclaved 4% thioglycolate, and 4 days later, these mice were anaesthetized with ketamine/xylazine and the peritoneal lavage was collected in DMEM. Cells were plated at 3 × 10^5^ cells/cm^2^ dish in DMEM containing penicillin (100 units/ml) and streptomycin (100 μg/ml). After 3 h, the floating cells (mostly red blood cells) were removed by washing with cold PBS and the adherent cells (macrophages) were used as needed.

### Cell culture

RAW264.7 cells (Cat # TIB-71) and MASMCs (Cat # CRL-2797) were purchased from American Type Culture Collection, and Cell Biologics Inc, respectively. RAW264.7 cells, MASMCs, and mouse peritoneal macrophages were cultured and maintained in DMEM containing 10% FBS, 100 units/ml penicillin, and 100 μg/ml streptomycin at 37 °C in a humidified 95% air and 5% CO_2_ atmosphere. HASMCs (Cat # ACBRI 716) that were originated from normal human descending aorta were obtained from Cell systems and cultured in medium 231 containing smooth muscle cell growth supplements, gentamicin (10 μg/ml), and amphotericin (250 ng/ml) at 37 °C in a humidified 95% air and 5% CO_2_ atmosphere. THP1 cells (Cat # TIB-202) were purchased from American Type Culture Collection and cultured in RPMI 1649 medium containing FBS (10%), penicillin (100 units/ml), streptomycin (100 μg/ml), and β-mercaptoethanol (50 μM) at 37 °C in a humidified 95% air and 5% CO_2_ atmosphere. The cells were growth-arrested overnight in basal medium without growth supplements and used unless otherwise indicated.

### Transfections

RAW264.7, MASMCs, HASMCs, and PMA-differentiated THP1 cells were transfected with scrambled or targeted siRNA at a final concentration of 100 nmoles using Lipofectamine 3000 transfection reagent according to the manufacturer’s instructions. After transfections, cells were recovered in complete medium for 24 h, growth-arrested overnight in serum-free medium and used as required.

### Quantitative reverse transcriptase-PCR

Total cellular RNA was isolated from aorta and peritoneal macrophages of CD-fed WT and CD or WD-fed ApoE^−/−^ and ApoE^−/−^:TRIM13^−/−^ mice as well as from MASMCs and RAW264.7 cells with appropriate treatments using TRIzol reagent as per the manufacturer’s instructions. A high-capacity complementary DNA reverse-transcription kit (Cat # 4387406, Applied Biosystems) was used for complementary DNA preparation. Quantitative reverse transcriptase-PCR was then performed on a 7300 Real-Time PCR system (Applied Biosystems) using SYBR Green Master Mix (Cat # A25741, Applied Biosystems) as per the manufacturer’s instructions. β-Actin was used as internal control. The PCR amplifications were examined using the 7300 Real-Time PCR system operated SDS version 1.4 program (https://www.thermofisher.com/us/en/home/technical-resources/software-downloads/applied-biosystems-7300-real-time-pcr-system.html) and Delta Rn analysis (Applied Biosystems).

### Western blotting

Cells with appropriate treatments were washed with PBS and scraped into RIPA buffer with inhibitors (PBS containing 1% Nonidet P40, 0.5% sodium deoxycholate, 0.1% SDS, 100 μg/ml PMSF, 100 μg/ml aprotinin, 1 μg/ml leupeptin, and 1 mM sodium orthovanadate) and lysed by sonication at 45% amplitude for 15 s with 10 s intervals for 2 min on ice (Branson Sonifier 450). To prepare aortic extracts, it was cleaned from perivascular fat, chopped, and minced in RIPA buffer using hand homogenizer and then sonicated at 45% amplitude for 15 s with 10 s intervals for 4 min on ice. Cell or tissue extracts were cleared by centrifugation at 12,000 rpm for 10 min at 4 °C and protein concentration was determined using Mico bicinchoninic acid kit (Thermo Fisher Scientific). Cell or tissue extracts containing equal amounts of protein from control and the indicated treatments were resolved by electrophoresis on 0.1% (w/v) SDS and 10% or 12% (w/v) polyacrylamide gels. The proteins were transferred electrophoretically onto a nitrocellulose membrane. After blocking in 5% (w/v) nonfat dry milk or 5% bovine serum albumin (BSA), the membranes were incubated with the appropriate primary antibodies at 1:1000 dilution overnight at 4 °C, followed by incubation with HRP-conjugated secondary antibodies at 1:5000 dilution for 1 h at room temperature (RT). After washing in Tris buffered saline containing Tween 20 for three times, the membranes were incubated with enhanced chemiluminescence detection reagent (Thermo Fisher Scientific) to detect the antigen-antibody complexes. The band intensities were quantified by densitometry using NIH ImageJ (https://imagej.net/ij/download.html) software.

### Pull-down assay

Cell and tissue extracts containing equal amount of protein from control and the indicated treatments were incubated with the indicated antibodies overnight at 4 °C, followed by incubation with protein A/G-Sepharose CL-4B beads for 3 h with gentle rocking at 4 °C. The beads were collected by centrifugation at 1000 rpm for 2 min at 4 °C and washed 3× with RIPA buffer and once with PBS. The immunocomplexes were released by heating the beads in 40 μl of 2× Laemmli sample buffer and analyzed by Western blotting for the indicated molecules using their specific antibodies.

### Proximity ligation assay

After appropriate treatments, peritoneal macrophages and MASMCs were fixed with 3.7% paraformaldehyde (PFA) for 15 min, permeabilized in 0.3% Triton X100 for 15 min and blocked with 3% BSA for 1 h. The cells were then incubated with mouse anti-TRIM13 (1:100) and rabbit anti-LXRα, LXRβ, SOCS1, or SOCS3 antibodies (1:100) overnight at 4 °C followed by incubation with goat anti-mouse secondary antibody conjugated with oligonucleotide PLA probe Plus and goat anti-rabbit secondary antibody conjugated with oligonucleotide PLA probe Minus, respectively. The cells were then incubated with a ligation solution for 30 min at 37 °C followed by a rolling-circle amplification of ligated oligonucleotide probes for 2 h at 37 °C. A fluorescently labeled complementary oligonucleotide detection probe was used to amplify the oligonucleotides conjugated to the secondary antibodies. After washing with wash buffer, the slides were mounted with a mounting media containing 4′,6-diamidino-2-phenylindole. PLA signals were examined using a Zeiss inverted fluorescence microscope (Axio Observer. Z1; x100/0.045NA), and the fluorescence images were captured using Zeiss AxioCam MRm camera and microscope operating image analysis software ZEN (Blue edition) V2.0 (https://asset-downloads.zeiss.com/catalogs/download/mic/8db1eb8d-7b2a-46e8-8427-f259dcfd1fb3/EN_quick-guide_ZEN-blue-edition_first-steps.pdf).

### Cholesterol efflux assay

RAW264.7 cells or MASMCs that were transfected with the indicated siRNA or peritoneal macrophages of CD or WD-fed ApoE^−/−^ and ApoE^−/−^:TRIM13^−/−^ mice were plated in 12-well plates at a density of 2 × 10^5^ cells/well. Cells were incubated with oxLDL (20 μg/ml) and [^3^H]-cholesterol (1 μCi/ml) for 24 h followed by extensive washings with PBS. Cells were then equilibrated in serum-free DMEM containing 0.2% fatty acid free-BSA for 2 h. After equilibration, the medium was replaced with fresh DMEM containing 0.2% fatty acid free-BSA and ApoA-I (10 μg/ml) or HDL (10 μg/ml) and incubation was continued for 6 h. In case of MASMCs and RAW264.7 cells, incubation was continued in the presence and absence of IL-1β (25 ng/ml) for 6 h. An aliquot of the efflux medium (100 μl) was removed for radioactivity determination. Cells were then rinsed with PBS, dried, and isopropanol was added for cholesterol extraction overnight at RT. An aliquot of the extract (100 μl) was used for radioactivity counting by LS6500 Multipurpose Scintillation Counter (Beckman Coulter). Cholesterol efflux was measured as % of total cellular radioactivity released into the medium.

### Foam cell formation assay

Peritoneal macrophages from CD or WD-fed ApoE^−/−^ and ApoE^−/−^:TRIM13^−/−^ mice or RAW264.7 cells, MASMCs, HASMCs, and PMA-differentiated THP1 cells that were transfected with the indicated siRNA, growth-arrested, treated with and without IL-1β (25 ng/ml) for 6 h were incubated with oxLDL (10 μg/ml) for 6 h at 37 °C. Cells were then fixed with 4% paraformaldehyde for 30 min, stained with Oil Red O for 15 min, and counter-stained with hematoxylin. Cell staining was observed under a Nikon Eclipse TS100 microscope with X20/0.4NA magnification and the images were captured using a Nikon Digital Slight DSL3 camera. After capturing the images, the Oil Red O stain was eluted by incubating the slides with isopropanol for 15 min at RT, and the optical density was measured at 500 nm in a SpectraMax 190 spectrophotometer (Molecular Devices). In the case of BODIPY staining, PFA fixed cells were stained with 2 μM BODIPY solution for 15 min and counterstained with 4′,6-diamidino-2-phenylindole, mounted with a cover slip using ProLong Gold antifade reagent and observed under a Zeiss inverted microscope (AxioObserver.Z1; X10/0.25 NA). The fluorescence images were captured using Zeiss AxioCam MRm camera and analyzed by ZEN (Blue edition) V2.0 (https://asset-downloads.zeiss.com/catalogs/download/mic/8db1eb8d-7b2a-46e8-8427-f259dcfd1fb3/EN_quick-guide_ZEN-blue-edition_first-steps.pdf) software.

### Aortic root cross sections

Aortic root cross sections were made from the hearts of CD-fed ApoE^−/−^ and 12 weeks of WD-fed ApoE^−/−^ and ApoE^−/−^:TRIM13^−/−^ mice for immunohistochemistry and immunofluorescence staining. Briefly, anesthetized mice were perfused with 4% PFA followed by PBS and the hearts were dissected out, fixed in 4% PFA overnight and then placed in OCT. Sequential 10 μm aortic root sections were cut from the point of appearance of the aortic valve leaflets with a Leica CM3050S cryostat machine (Leica Biosystems).

### Oil Red O staining

The aortic root sections from 12 weeks of WD-fed ApoE^−/−^ and ApoE^−/−^:TRIM13^−/−^ mice were fixed with 10% formaldehyde solution, washed with PBS, rinsed with 60% isopropanol, and stained with Oil Red O for 15 min followed by counterstaining with haematoxylin. The sections were observed under a Nikon Eclipse 50i microscope with X4/0.1 NA, and the images were captured with a Nikon Digital Sight DS-L1 camera.

### Enface staining

Aortas were excised from 12 weeks of WD-fed ApoE^−/−^ and ApoE^−/−^:TRIM13^−/−^ mice, and perivascular fat was removed carefully with scissors, opened longitudinally, and fixed in 4% PFA-sucrose solution overnight at RT. The aortas were then washed with PBS three times, fixed in 70% ethanol for 5 min, stained with Sudan IV for 10 min and washed once with 70% ethanol and once with PBS at RT. The pictures were taken using Nikon D7100 camera, and the percent surface area occupied by the lesions was measured using the NIH ImageJ.

### Immunofluorescence staining

The mouse aortic root sections were fixed with acetone/methanol (1:1), permeabilized with Triton X100, and blocked with 3% BSA containing 5% goat serum. The human nonstenotic and stenotic coronary artery sections were deparaffinized with xylene and then treated with antigen masking solution for 15 min at 95 °C. The sections were then permeabilized with 0.5% Triton X100 for 15 min and blocked with normal goat serum. After blocking, sections were incubated with the indicated antibodies in combination with SMMHC or CD68, specific markers for SMC and macrophages, respectively, at 1:100 dilution, followed by incubation with Alexa Fluor 488–conjugated goat anti-mouse and Alexa Fluor 568–conjugated goat anti-rabbit secondary antibodies at 1:500 dilution. The sections were washed with PBS, counterstained with Hoechst, mounted with a cover slip using ProLong Gold antifade reagent and observed under a Zeiss inverted microscope (AxioObserver.Z1; X10/0.25 NA). The fluorescence images were captured using Zeiss AxioCam MRm camera and analyzed by ZEN (Blue edition) V2.0 software.

### Statistics

All the experiments were repeated three times. Normality of the data was checked using Anderson-Darling Normality Test, and F-test was used to assess the equality and group variance. The treatment effects were analyzed by student *t* test for two group comparisons and one-way ANOVA followed by Bonferroni post hoc test for multiple group comparisons. All the statistical tests were performed using GraphPad Prism v 8.00 (https://www.graphpad.com) software. In the case of *in vivo* experiments, a minimum of seven mice for each gender was included. Since we did not observe statistical differences between male and female mice in our experiments, we did not present the results in a gender-specific manner. In case of human tissue section staining, the sections from five different individuals for each lesion grade were used. The data are presented as mean ± SD and the *p* <0.05 were considered statistically significant.

## Data availability

The datasets used and/or analyzed during the current study are available from the corresponding author on reasonable request.

## Supporting information

This article contains supporting information.

## Conflict of interest

The authors declare that they have no conflict of interest with the contents of this article.
