# Supplementary figures and images for: TRIM13 reduces cholesterol efflux and increases oxidized LDL uptake leading to foam cell formation and atherosclerosis
Source: J Biol Chem. 2024 Mar 25;300(5):107224. doi: 10.1016/j.jbc.2024.107224 (PMC11053335; doi:10.1016/j.jbc.2024.107224)

Figure S1

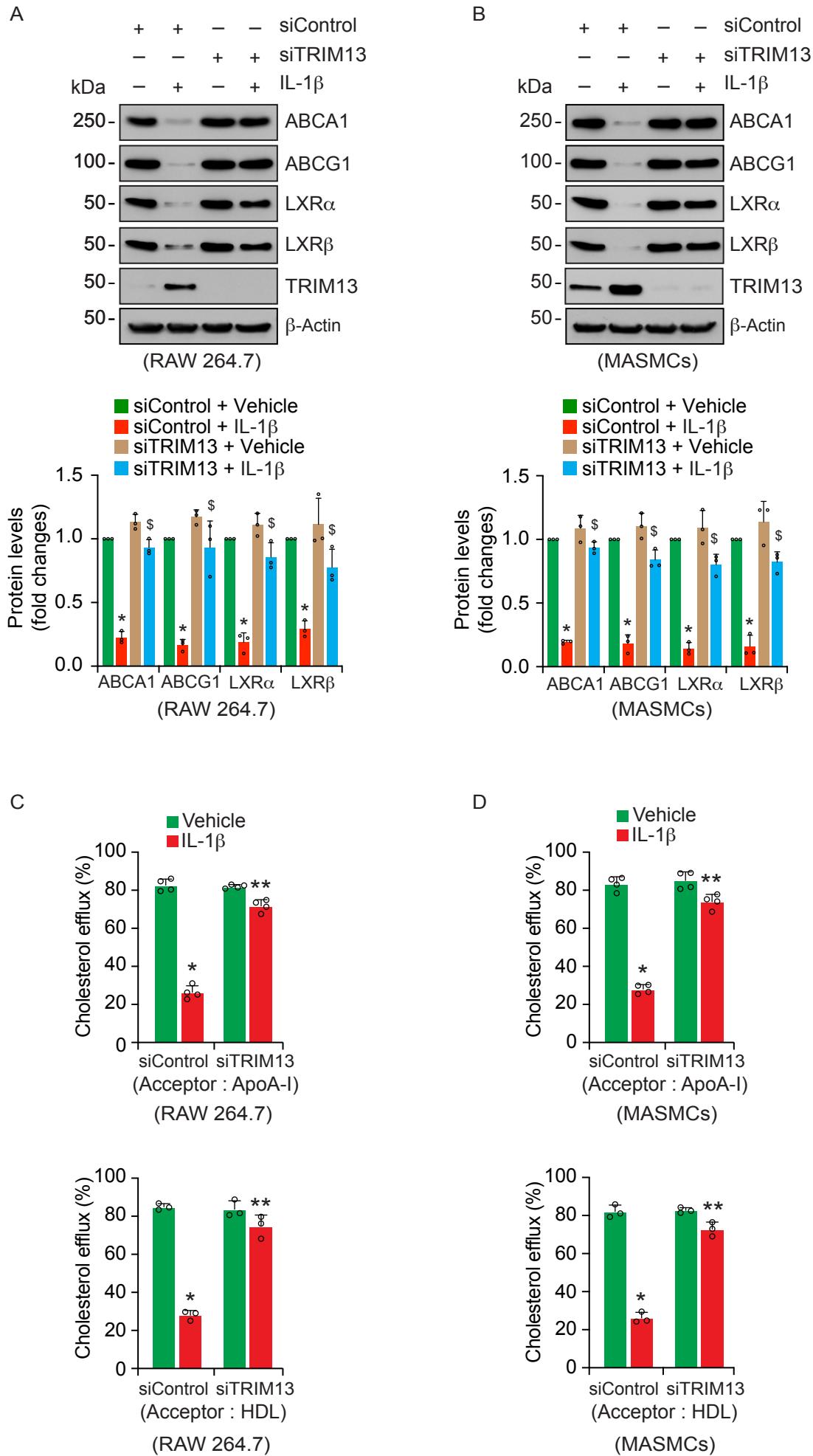

Supplement: Supporting Figure S1 [file mmc1.pdf]

Figure S2

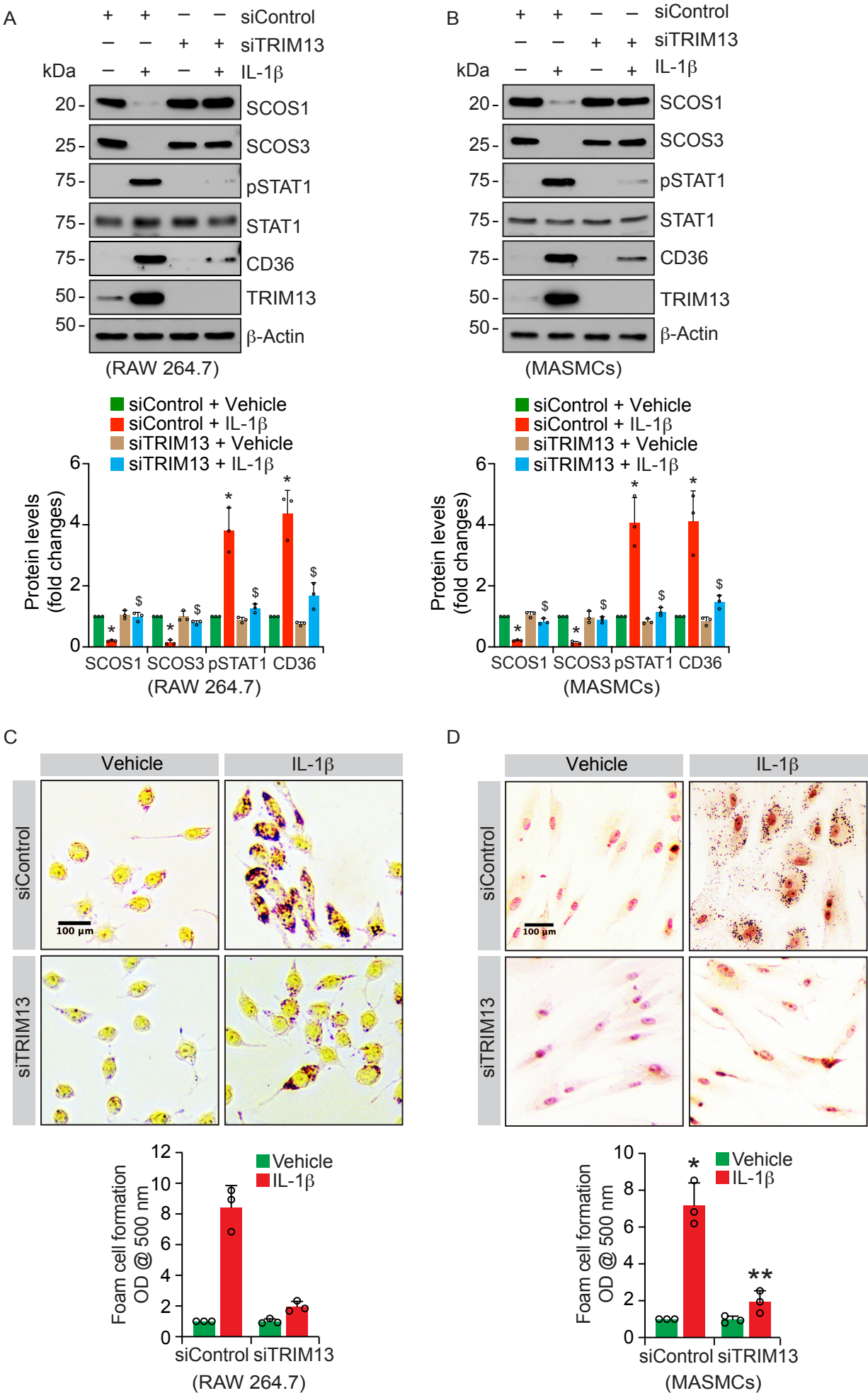

Supplement: Supporting Figure S2 [file mmc2.pdf]

Figure S3

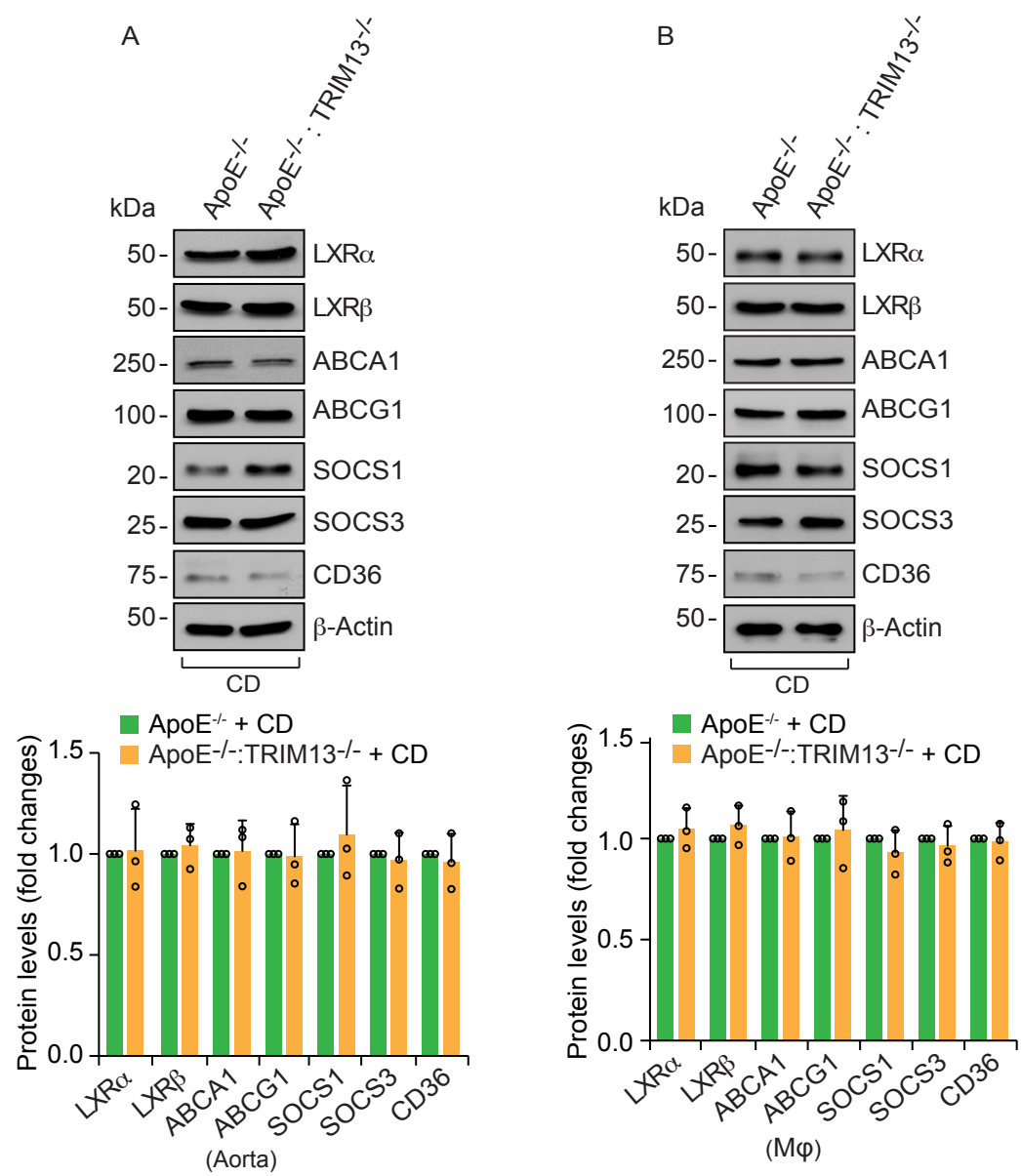

Supplement: Supporting Figure S3 [file mmc3.pdf]

Figure S4

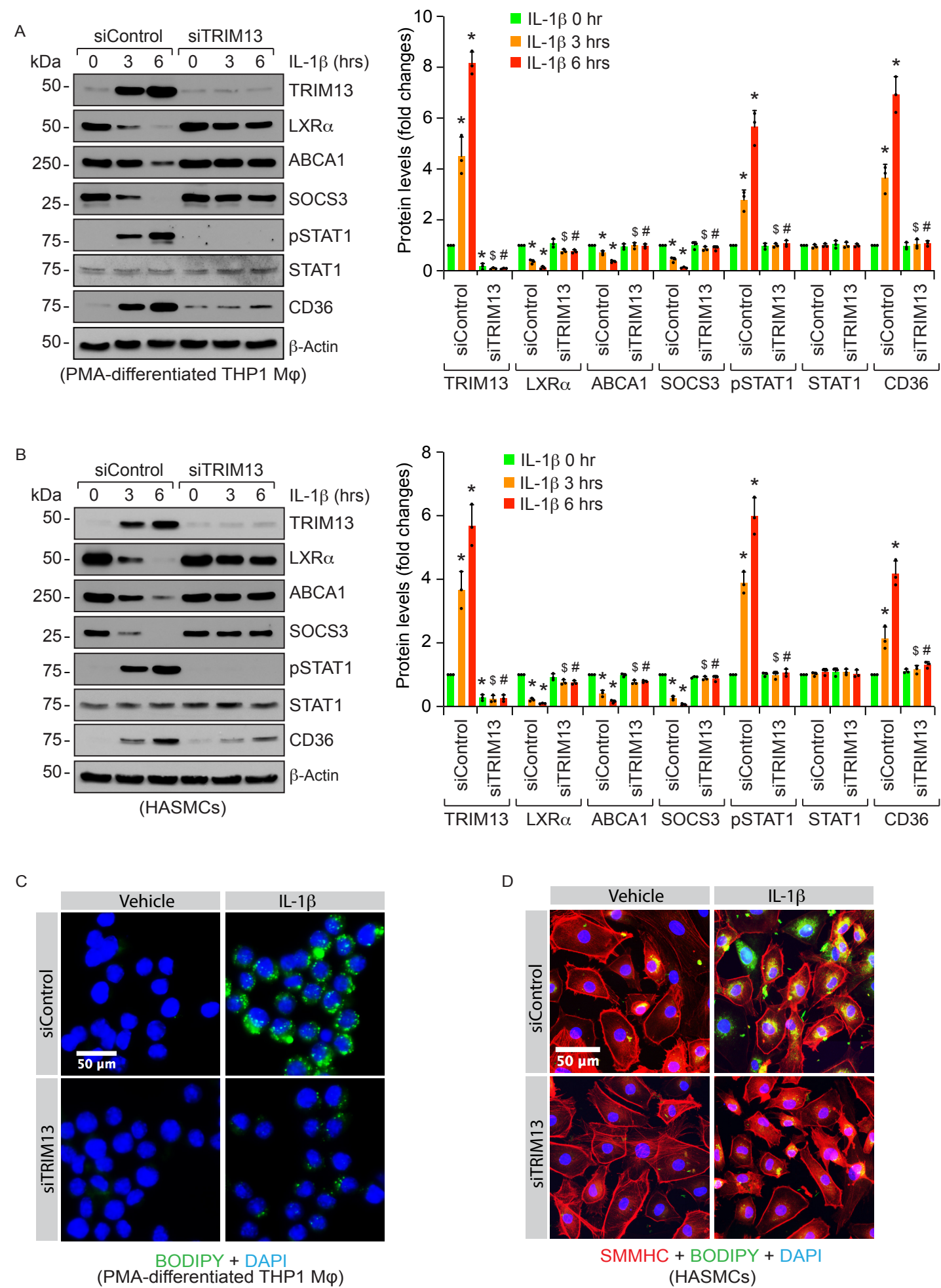

Supplement: Supporting Figure S4 [file mmc4.pdf]
