# Supplementary material for: TRIM13 reduces cholesterol efflux and increases oxidized LDL uptake leading to foam cell formation and atherosclerosis
Source: J Biol Chem. 2024 Mar 25;300(5):107224. doi: 10.1016/j.jbc.2024.107224 (PMC11053335; doi:10.1016/j.jbc.2024.107224)
Supplement: Supporting Table S1 [file mmc5.doc]

**Table S1. List of primers**

| **Gene** | **Forward primers 5′ → 3′** | | **Reverse primers 5′ → 3′** |
| --- | --- | --- | --- |
| **qRT-PCR primers** | | | |
| March6 | CACTACTGCTCTGTGGCACA | | ACCGGCGGATTTACCCATTT |
| Gp78 | AAAGGGCCTCCTCATTCTGC | | GAAGAGGCCTGTCCCAGTTC |
| Dtl | GCACAGCAAGTAACCCAAGC | | CACACACATGCAGCAGCTTT |
| Hace1 | GCTGTCCTGAGTGCAGATGT | | GCCAGGTGTTCAAGCCTTTG |
| Hectd1 | GCCTGTTCTGTAGCCACACT | | CAGAAGAATGCAGATGCCGC |
| Nedd4 | TGGAACTCGGTTCTTCACGG | | GGTTCATGTGTACGGCAGGA |
| Siah1 | CAGCCACGCCATCATGAATG | | ATGCCGCCAGAAGTTAAGCT |
| Smurf1 | ACACAGCCAGACCCATGATG | | GAAGCACAGTGTCGGGAAGA |
| Trim63 | GACTTTTCCAGCTGCTCCCT | | TCTGGAGGTCGTTTCCGTTG |
| TRIM13 | CTGCCACATTGTTCAGTGCC | | TTTACAGGAGCAGCGGATGG |
| Idol | TGTGGAATGAGGGGAAACGG | | GACCACACCTCTTCCGTGAG |
| Cbl | CCACAGAAGCCAGAAGAGGG | | ATCAAGGAAACAGCACTGGC |
| Herc2 | AGACCAGCCGCTCTGTAGTA | | CACTGCCTTGCTCTGACAGA |
| Huwe1 | TACCAGAGTCCCCACGAGAG | | TCCGCCGGAGTTCTCTCTTA |
| Lnx1 | TACCAGAGTCCCCACGAGAG | | TCCGCCGGAGTTCTCTCTTA |
| Rnf20 | ACGGTCATCTTTGCGTTCCT | | GCTCGCCATCTAGCATCCAT |
| Peli1 | ATGGACATGGCCGCAGTTTA | | TCATATGTGAGCGCAGTCCC |
| COP1 | TGCAGGTGTGTGTATGGGTC | | AATGGTCTGCACTGTCCAGG |
| Pja2 | TTGGCCACCTCAACGTCTAC | | AGTGCAGTGATGGGGAATGG |
| Ubr2 | AGTCCCTGGAGCCAGACTAG | | GGGAGTCTCCTCTGCTCTCA |
| LXRα | TGCTGATGGCAATGAGCAGA | | CTGAAGCGGCAAGAAGAGGA |
| LXRβ | GAAGCCTTTGCAGCCTTCAC | | AGCTACTCCCAGGCTTCTGA |
| ABCA1 | CATTTCGAAGGAGACAAACATGTCA | | CATGGCTTTATTCGGAAAGTGGACC |
| ABCG1 | AAGCCAGAAAGCAGGGACTC | | CCTGCCTCCTCTTCTACCCT |
| SOCS1 | gcatccctcttaacccggtac | | aaatgaagccagagaccctcc |
| SOCS3 | gagttttctctgggcgtcctcctag | | tggtactcgcttttggagctgaa |
| CD36 | CATGTCGCAATAGCTTGGCC | | GACGTGGCAAAGAACAGCAG |
| **Genotyping primers** | | | |
| ApoE^-/-^ | | Common: 5′-gcctagccgagggagagccg-3′  Wild type reverse: 5′-TGTGACTTGGGAGCTCTGCAGC-3′  Mutant reverse: 5′-GCCGCCCCGACTGCATCT-3′ | |
| Trim13^-/-^ | | Common: 5′-CCCGAAGTAAAGAGCCTATTCTGTC-3′  Wild type reverse: 5′-GATCCCACAAATCAGCTGCATATC-3′  Mutant reverse: 5′-ACTACCATCATGAGCAGCAGATAG-3′ | |
