# Supplementary material for: TRIM13 reduces cholesterol efflux and increases oxidized LDL uptake leading to foam cell formation and atherosclerosis
Source: J Biol Chem. 2024 Mar 25;300(5):107224. doi: 10.1016/j.jbc.2024.107224 (PMC11053335; doi:10.1016/j.jbc.2024.107224)
Supplement: Supporting information [file mmc6.doc]

**SUPPORTING INFORMATION**

**TRIM13 reduces cholesterol efflux and increases oxidized LDL uptake leading to foam cell formation and atherosclerosis**

Suresh Govatati^1#^, Raj Kumar^1#^, Monoranjan Boro^1#^, James G. Traylor Jr.^2^, A. Wayne Orr^2^, Aldons J. Lusis^3^, Gadiparthi N. Rao^1^

^1^ Department of Physiology, University of Tennessee Health Science Center, Memphis, TN, 38163, USA

^2^ Department of Pathology, Louisiana State University Health Science Center, Shreveport, LA, 71103, USA

^3^ Division of Cardiology, Department of Medicine, University of California, Los Angeles, CA, 90095, USA

^#^ The first three authors contributed equally to this work.

**Running title:** TRIM13 role in diet-induced atherosclerosis

**Address correspondence to:**

Gadiparthi N. Rao, Ph.D.

Department of Physiology

University of Tennessee Health Science Center

71 S Manassas Street

Memphis, TN 38163

Phone: 901-448-7321

Email: rgadipar@uthsc.edu

**SUPPORTING FIGURE LEGENDS**

**Figure S1. Depletion of TRIM13 restores cholesterol efflux from IL-1β-induced downregulation:** A and B, RAW 264.7 cells or MASMCs were transfected with siControl or siTRIM13 (100 nmoles) and 36 hrs later cells were quiesced, treated with and without IL-1β (25 ng/ml) for 6 hrs and cell extracts were prepared and analyzed by Western blotting for the indicated proteins. Cell extracts were also analyzed by Western blotting for TRIM13 and β-actin levels to show the efficacy of siRNA on its on-target and off-target molecules (n = 3). C and D, All the conditions were same as in panels A and B except that cells were assayed for cholesterol efflux using ApoA-I or HDL as an acceptor (n = 3). The bar graphs represent Mean ± SD values of three independent experiments. **p*<0.01 versus siControl + vehicle; ^$^*p*<0.01 versus siControl + IL-1β.

**Figure S2. Depletion of TRIM13 inhibits IL-1β-induced foam cell formation:** A and B, RAW 264.7 cells or MASMCs that were transfected with siControl or siTRIM13 (100 nmoles) were quiesced, treated with and without IL-1β (25 ng/ml) for 6 hrs, and cell extracts were prepared and analyzed by Western blotting for the indicated proteins. Cell extracts were also analyzed for TRIM13 and β-actin levels to show the efficacy of siRNA on its on-target and off-target molecules (n = 3). C and D, All the conditions were same as in panels A and B except that cells were assayed for foam cell formation using Oil Red O staining (n = 3). The bar graphs represent Mean ± SD values of three independent experiments. Scale bar is 100 μm. **p*<0.01 versus siControl + vehicle; ^$^*p*<0.01 versus siControl + IL-1β.

**Figure S3. Lack of differences in TRIM13 downstream effectors levels between ApoE^-/-^ and ApoE^-/-^:TRIM13^-/-^ mice on CD:** A and B, Protein extracts from aortas (A) and peritoneal macrophages (B) of ApoE^-/-^ and ApoE^-/-^:TRIM13^-/-^ mice fed with CD were analyzed by Western blotting for the indicated proteins using their specific antibodies (n = 3). The bar graphs represent Mean ± SD values of three independent experiments.

**Figure S4. Effects of IL-1β on TRIM13 and its downstream effectors and foam cell formation in human cells:** A and B, PMA-differentiated THP1 cells (A) and HASMCs (B) were transfected with siControl or siTRIM13 (100 nmoles), quiesced, treated with and without IL-1β (25 ng/ml) for indicated time periods, cell extracts were prepared and analyzed by Western blotting for the indicated proteins. Cell extracts were also analyzed for TRIM13 and β-actin levels to show the efficacy of siRNA on its target and off-target molecules (n = 3). C and D, All the conditions were same as in panels A and B except that cells were assayed for foam cell formation. PMA-differentiated THP1 cells were stained for BODIPY whereas HASMCs were coimmunostained for BODIPY along with SMMHC (n = 3). Scale bar is 50 μm in panels C and D. The bar graphs represent Mean ± SD values of three independent experiments. **p*<0.01 versus siControl + vehicle; ^$^*p*<0.01 versus siControl + IL-1β (3 hrs); ^#^*p*<0.01 versus siControl + IL-1β (6 hrs).
